# Securing Group Patient Communication in 6G-Aided Dynamic Ubiquitous Healthcare with Real-Time Mobile DNA Sequencing

**DOI:** 10.3390/bioengineering10070839

**Published:** 2023-07-15

**Authors:** Tuan-Vinh Le

**Affiliations:** 1 Bachelor’s Program of Artificial Intelligence and Information Security, College of Science and Engineering, Fu Jen Catholic University, New Taipei 24206, Taiwan; 155315@mail.fju.edu.tw; 2 Bachelor’s Program of Medical Informatics and Innovative Applications, College of Science and Engineering, Fu Jen Catholic University, New Taipei 24206, Taiwan

**Keywords:** 6G technology, third-generation sequencing (TGS), DNA-reading biosensor, ubiquitous healthcare (U-healthcare), patient-centric care, internet of living things (IoLT), dynamic group patient communication, sequencing-device-based single sign-on (SD-SSO), biometric authentication, elliptic curve cryptography (ECC)

## Abstract

(1) Background: With an advanced technique, third-generation sequencing (TGS) provides services with long deoxyribonucleic acid (DNA) reads and super short sequencing time. It enables onsite mobile DNA sequencing solutions for enabling ubiquitous healthcare (U-healthcare) services with modern mobile technology and smart entities in the internet of living things (IoLT). Due to some strict requirements, 6G technology can efficiently facilitate communications in a truly intelligent U-healthcare IoLT system. (2) Research problems: conventional single user–server architecture is not able to enable group conversations where “multiple patients–server” communication or “patient–patient” communication in the group is required. The communications are carried out via the open Internet, which is not a trusted channel. Since heath data and medical information are very sensitive, security and privacy concerns in the communication systems have become extremely important. (3) Purpose: the author aims to propose a dynamic group-based patient-authenticated key distribution protocol for 6G-aided U-healthcare services enabled by mobile DNA sequencing. In the protocol, an authenticated common session key is distributed by the server to the patients. Using the key, patients in a healthcare group are allowed to securely connect with the service provider or with each other for specific purposes of communication. (4) Results: the group key distribution process is protected by a secure three-factor authentication mechanism along with an efficient sequencing-device-based single sign-on (SD-SSO) solution. Based on traceable information stored in the server database, the proposed approach can provide patient-centered services which are available on multiple mobile devices. Security robustness of the proposed protocol is proven by well-known verification tools and a detailed semantic discussion. Performance evaluation shows that the protocol provides more functionality and incurs a reasonable overhead in comparison with the existing works.

## 1. Introduction

**Figure 1:**
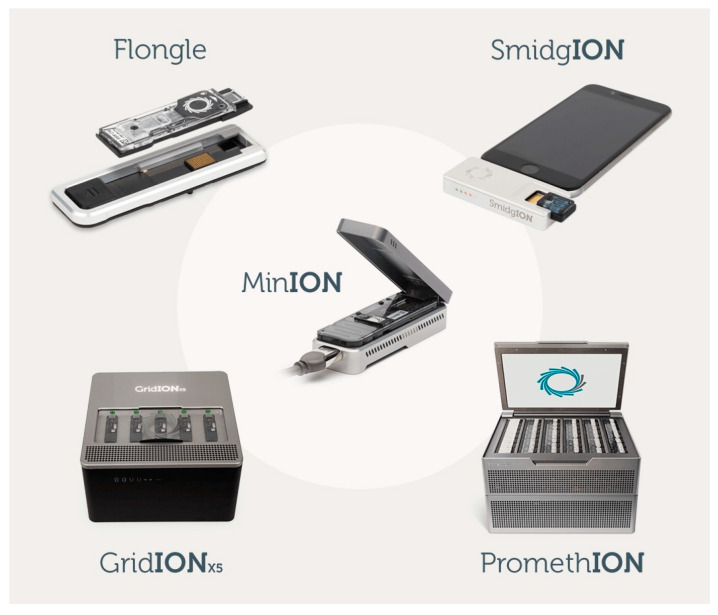
New innovative sequencers of the Oxford Nanopore [8].

Third-generation sequencing (TGS) provides services with long deoxyribonucleic acid (DNA) reads and super short sequencing time [1,2,3]. In this technique, since single DNA molecules are sequenced directly, the sequencing time is reduced to a few hours, and even real-time data analysis process is enabled. In addition, TGS-based sequencers can be miniaturized while its DNA-reading biosensors are placed on the body to monitor human health and vital signs via blood, sweat, saliva, tissue, etc. [3]. This enables an onsite mobile DNA sequencing solution for facilitating ubiquitous healthcare (U-healthcare) services with modern mobile technology and smart systems in the internet of living things (IoLT) [3,4]. For instance, as shown in Figure 1, the SmidgION sequencer is a tiny device designed by the Oxford Nanopore [5] to be run on mobile devices (e.g., smart phones) using small batteries and apps [3]. The biosensors load biological samples into the sequencer, and the genomic data (e.g., FAST5 file, FASTQ file, or TXT file [5]) along with its analytical results are produced, building a sort of “lab-on-a-chip (LOC)” system [3,5,6,7]. Therefore, medical providers can rapidly screen for new viruses, paving a way for further discovering the IoLT. The researchers can also obtain onsite DNA sequences for specific end-to-end analysis. The U-healthcare is directly concerned with patient-centric therapies. To this end, a real-time mobile DNA sequencing service is completely a good fit as it can provide personalized treatments and holds promise for precision medicine research.

Due to its excellent mobility, high operating frequency, high data transfer rate, and super low end-to-end communication delay, 6G mobile technology is attracting much attention in various application fields [9,10,11]. Strict requirements of 6G, which cannot be achieved by 5G, were particularly introduced for the healthcare sector, including an operating frequency of ≥1 THz, data transfer rate of ≥1 Tbps, communication delay of ≤1 ms, mobility of ≥1000 km/h, reliability of $10^{-9}$, and a wavelength of ≤300 µm [10]. Due to such advances, 6G can efficiently support artificial intelligence (AI) functionalities [12] with seamless communications. As a matter of fact, it has certain advantages in establishing a truly intelligent U-healthcare IoLT system enabled by real-time mobile NDA sequencing techniques and advanced medical analysis. Patients and healthcare providers are allowed to communicate with each other in a reliable and high-speed network environment, possibly sharing large files or a huge amount of data.

### 1.1. Research Problems

Apart from individual services, healthcare providers may provide some special treatments for groups of patients (e.g., family). These patients may have similar diseases, signs, or symptoms. They can also be persons those who need similar procedures in the healthcare processes or medical treatments. Traditional single user–server architecture is not able to provide such group conversations where “multiple patients–server” communications and “patient–patient” communications are required.

The communications are carried out via the open Internet, which is not a trusted channel. Because heath data and medical information are very sensitive, security and privacy concerns in the communication systems have become extremely important. Cyber criminals may perform various attacks that can steal personal information of patients, violate user privacy, or disrupt services (e.g., impersonation attacks). During communication, care providers (e.g., medical professionals, physicians, doctors, etc.) also need to verified as a legitimate entity to avoid possibly fraudulent services or fake behaviors.

The U-healthcare services may be provided by different institutions, including hospitals, clinics, etc.; the number of services (e.g., hematologist, cardiologist, gastroenterologist, etc. [13]) is increasing over time. Therefore, the traditional single-server system model would be unable to satisfy the demand of users once they wish to enjoy massive medical services. When using services from multiple providers, remembering massive amounts of credentials (especially user passwords) for the login will certainly induce inconvenience and directly affect the efficiency of communications. In these systems, how to alleviate computational overhead and communicational overhead is also an important concern that needs to be considered.

### 1.2. Goals and Contributions

This paper proposes a dynamic group-based patient-authenticated key distribution protocol for 6G-aided U-healthcare services enabled by real-time mobile DNA sequencing. In the protocol, an authenticated common session key is distributed by the server to the patients. Using the key, patients in a healthcare group are allowed to securely connect with the service provider or with each other for specific purposes of communication. The author aims to introduce a protocol that achieves multiple innovative functionalities, high security robustness, and reasonable communication overhead. The main contributions of the paper are presented as follows.

(1) This work is the first to introduce 6G-assisted group-based U-healthcare services enabled by a real-time DNA sequencing technique constructed in IoLT environments. A patient-grouping solution helps in accelerating service communications and achieving better medical-centered services. With the assistance of 6G technology, onsite sequencing data produced by a portable TGS-based sequencer (connected to a patient’s mobile device) is transmitted to the server in a real-time manner for further healthcare processes. Thereafter, the server shares analytical results and related medical information with the patients. These procedures are secured by common group keys generated by the proposed protocol. The server is also allowed to trace the users based on their registered information for achieving a truly patient-centric service.
(2) In the proposed protocol, a sequencing-device-based single sign-on (SD-SSO) function is introduced for the first time. Patients are allowed to store a single set of credentials (registered with multiple servers) on their DNA sequencers directly. Due to the SSO property, the patients only need to login to the system once per session to communicate with multiple providers. In addition, the proposed SD-SSO function is designed without the participation of a third-party center, which can reduce communication overhead and address the risk of adversaries hacking into the registration center and compromising all servers.
(3) A three-factor authentication mechanism is enabled in the protocol through the integration of password (the first factor), sequencing device (the second factor), and biometrics (the third factor). Lacking only one of the three factors will result in failure of the authentication. In this way, better patient privacy and perfect forward secrecy of group keys are assured for securing U-healthcare communications. In the protocol, patient password and patient biometrics are changeable, which further enhances the security robustness.
(4) The author introduces dynamic U-healthcare services enabled by a time-bound function. In this design, different services of a provider or multiple healthcare processes in a single service can be allotted in respective time ranges in accordance with specific requests. This solution makes providers flexibly adjust service time in order to provide more efficient medical processes as well as more convenient treatments for different kinds of patients. Controlling such access to the services using the time bounds can also address possible bottleneck issues where the services are requested at the same time by massive patients. Furthermore, a fast synchronizable key-derivation procedure is provided, which can rapidly reset communication keys for addressing desynchronization problems that could possibly occur in such a dynamic environment.

### 1.3. Paper Organization

The remainder of this article is structured as follows. Section 2 presents related works of the proposed protocol. Some technical preliminaries used in the work are provided in Section 3. In Section 4, the problem formulation describes the architecture model and formal security model of the proposed work. Section 5 details the design of the proposed protocol. Security evaluation and performance analysis of the proposed protocol are provided in Section 6 and Section 7, respectively. The author concludes the proposal and discusses some of his future research works in the last section of the article.

## 2. Related Works

### 2.1. 5G, 6G, and U-Healthcare

In many countries, 5G mobile technology has been successfully developed and deployed as an enabler for supporting various sorts of networks and diverse applications [14]. However, in the era of digital transformation and emerging smart internet of things (IoT) applications, 5G needs some more advances to improve service delivery and business [15]. Moreover, 5G has some drawbacks and limitations in terms of functionalities in healthcare sector; for instance, it cannot provide holographic communication for medical applications due to its lower data rate [9,16]. To this end, 6G was introduced to fully address escalating technical demands, e.g., remote robotic surgery or other truly intelligent healthcare services enabled by the Intelligent Radio (IR) technique [17]. It achieves an ultra-high bandwidth (three times higher than that of 5G [18,19]) and a highly dynamic environment with a terahertz (THz) signal [18]. Therefore, 6G offers an ultra-high data transfer rate for revolutionizing U-healthcare communications. It is also fully backed by satellite [20], which completely facilitates ubiquitous care activities in medical networks at every geographical location. This article introduces a construction of 6G wireless technology for a time-bound-enabled DNA-based group healthcare application via IoLT-based biosensor networks. In addition, to the best of the author’s knowledge, this is the first work to address security and privacy issues in a dynamic U-healthcare communication environment.

### 2.2. User Authentication and Key Negotiation Solutions

User authentication and key agreement solutions were discussed in many previously published works. Deebak and Al-Turjman [21] introduced a patient authentication scheme used in healthcare systems with cloud services; it overcame several security challenges that had been not successfully addressed in the protocol of Chiou et al. [22], e.g., lost device attacks or server impersonation attacks. Wang et al. [23] also proposed an improved key agreement mechanism for wireless body area networks (WBANs) that resolved some similar issues of Farash et al. [24]’s work. Kumar et al. [25] discussed a single-factor password-based patient authentication solution for cloud-based healthcare systems in the internet of medical things (IoMT). A two-factor data authentication scheme with access control was proposed by Gupta et al. [26] for an industrial healthcare infrastructure. Alam and Kumar [27] designed a session key establishment protocol for ensuring confidentiality of IoMT-based communications in COVID-19 and future pandemic scenarios. In addition, Thakare and Kim [28] discussed another two-factor cryptographic approach for user authentication in IoT networks, and Yu et al. [29] introduced a biometrics-based multi-server user authentication and key agreement mechanism using extended chaotic maps. Wong et al. [30] introduced a three-factor identification model applied to 5G-enabled e-health environments with multi-server architecture. However, Le and Hsu [31] indicated that biometrics noise had not been discussed and resolved in Wong et al. [30]’s work, which always makes the authentication procedure incorrect. Le and Hsu [31] then discussed various solutions [32] (error-correcting codes, fuzzy extractor, biohash function, etc.) for remedying this issue and proposed an improved protocol for securing communications in group e-health services. The author found the protocol of Le and Hsu [31] is not robust against stolen smart-card attacks as adversaries can obtain patients’ passwords in unmasked forms using the power analysis method [33]. Another design of lightweight group key agreement presented by Harn et al. [34] exploited some basic cryptographic operations and explained its potentials in several application networks. Based on principles of elliptic-curve cryptography (ECC), Tselikis et al. [35] also introduced an group key distribution scheme that provided privacy protection for communications. Both Harn et al. [34] and Tselikis et al. [35] did not include either biometric authentication function or three-factor authentication solutions in their designs. Meshram et al. [36] proposed a remote user password-based key negotiation scheme for application in smart cities based on smart cards and extended chaotic maps. Nevertheless, the service provider in Meshram et al. [36]’s scheme has to update a dynamic parameter in the database before each authentication is completed. This would sometimes result in unexpected desynchronization problems in the system. Based on the author’s observation, although Thakare and Kim [28] and Meshram et al. [36] achieved user anonymity in their works, both are not able to assure user untraceability. Communicated transcripts in their proposed schemes contain fixed parameters that give adversaries opportunities to trace users’ identities. Le [37] recently introduced a cross-server-authenticated patient key exchange protocol for U-healthcare in IoLT networks. Apart from its security robustness, Le [37]’s protocol cannot provide truly patient-centric services, as the server does not store any information of patients after its registration procedure finishes. In the registration phase of Le [37]’s approach, some credentials of the patients are stored in a single mobile entity, which cannot make U-healthcare services available on multiple devices. Furthermore, none of the above works discussed dynamic healthcare communication in group-based services.

## 3. Preliminaries

This section discusses some important technical aspects and mathematical preliminaries employed in the proposed approach, including sequencing biosensor technology, the biohash function, the time-bound function, and security complexity assumptions.

### 3.1. Sequencing Biosensor Technology

Second-generation sequencing (SGS) techniques, also known as next-generation sequencing (NGS) techniques, enable the process where millions of short deoxyribonucleic acid (DNA) fragments are sequenced in parallel [38]. Nevertheless, SGS comes with some drawbacks, including short read lengths and nonportability of the sequencers. In recent years, innovative healthcare services and medical research have required longer reads and shorter sequencing times, which led to the advent of TGS [3] and fourth-generation sequencing (FGS) [39]. From TGS, single DNA molecules are sequenced directly, reducing processing time from a few days to a few hours and enabling real-time analysis with sequence-based ultrarapid pathogen identification [3]. Sequencing devices can be miniaturized (for instance, SmidgION sequencer), and built-in DNA-reading biosensors on each tiny TGS-based sequencer can collect biological samples for monitoring human health and vital signs. In the proposed protocol, besides the sequencing function, the sequencer also serves as a token that stores user credentials used for authentication process, enabling service availability on multiple mobile devices including smart phones, smart tablets, etc. It is employed as the second authentication factor (something you have) in the proposed approach.

### 3.2. Biohash Function

As we know, biometric samples are enrolled via a noisy channel. The input biometrics samples in each authentication session are not identical; as a result, it causes false positive errors of the authentication. To this end, the biohash function can map the individuals’ biometrics to specific binary strings and effectively tolerate noise [32]. Security of the biohash function is similar to conventional one-way hash functions [31]. The function also resolves the efficiency issue, which is a drawback of some related ideas, for instance, fuzzy extractor [32].

**Definition 1.**  *Given a biohash function $h_{bio}$, the original biometrics $B_{i}$, and the newly input biometrics $B_{i}^{\prime }$ of an individual, it is inferred that $B_{i}$ is different to $B_{i}^{\prime }$, but the difference between them is within a certain threshold. Due to the property of $h_{bio}$, we can achieve $h_{bio}\left(B_{i}\right)=h_{bio}\left(B_{i}^{\prime }\right)$*.

### 3.3. Time-Bound Function

**Definition 2.**  *Given three time points $t,t_{1},t_{2}$ ∈ {*1*, *2*, …, z} and two values $p=h^{t_{1}-1}(▢)$ and $q=h^{z-t_{2}}(△)$, where h is a one-way hash function and “▢, △ ” denotes some arbitrary parameters, a value $w=h(h^{t-t_{1}}(p)||h^{t_{2}-t}\left(q\right))$ is computable if and only if t satisfies $t_{1}\leq t\leq t_{2}$. Note that z may be 24 (h), 1440 (min), or some value specifying the time of a single day. z may also be set for multiple days or more, based directly on time allocations of specific services and on security level of systems.*

### 3.4. Complexity Assumptions

The ECC is employed in the proposed approach. It is an asymmetric cryptosystem that offers better performance with smaller key space considering the same security level compared with the traditional ones [37]. Therefore, the ECC system is completely suitable for mobile communications in IoLT networks. In the proposed work, the author employs three security assumptions of the ECC including the elliptic curve discrete logarithm problem (ECDLP), the elliptic curve computational Diffie–Hellman problem (ECCDHP), and the elliptic curve factorization problem (ECFP). Suppose there is an elliptic curve $Ep\left(a,b\right):y^{2}=x^{3}+ax+b(mod\;p)$ over a finite field *Fp* with a basic point $G_{\left(x,y\right)}\in E_{p}$; the assumptions are defined as follows.

**Definition 3.**  *The ECDLP is to find the scalar* *$k\in Z_{p}$ such that $K_{\left(x,y\right)}=k\cdot G_{\left(x,y\right)}$, given $G_{\left(x,y\right)},K_{\left(x,y\right)}\in E_{p}$.*

**Definition 4.**  *The ECCDHP is to find the point $s\cdot t\cdot G_{\left(x,y\right)}\in E_{p}$, given $s,t\in Z_{p}$ and $G_{\left(x,y\right)},s\cdot G_{\left(x,y\right)},t\cdot G\in E_{p}$.*

**Definition 5.**  *The ECFP is to find two points $s\cdot G_{\left(x,y\right)},t\cdot G_{\left(x,y\right)}\in E_{p}$, given $s,t\in Z_{p}$ and $G_{\left(x,y\right)},[s+t]\cdot G_{\left(x,y\right)}\in E_{p}$.*

## 4. Problem Formulation

This section discusses in details system model of the proposed approach along with some well-known adversarial capabilities. A well-known security model is also formulated based upon the rule of the protocol. Main cryptographic functions and notations used in the work are tabulated in Table 1.

### 4.1. System Model and Adversarial Capabilities

**Figure 2:**
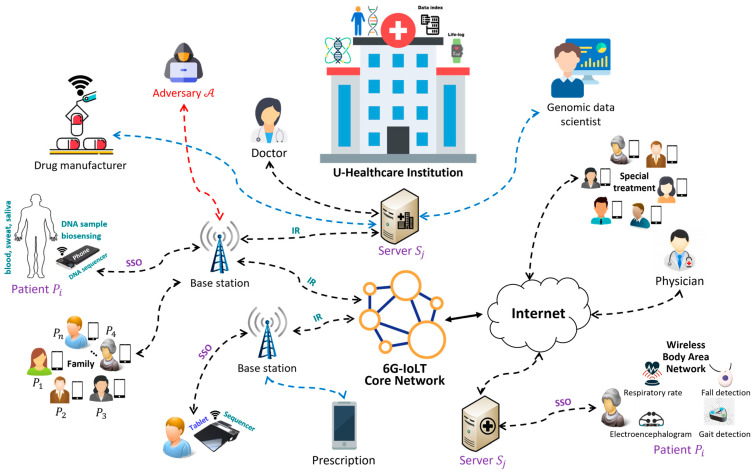
Architecture model of the proposed protocol.

As shown in Figure 2, the main communicating entities in the system include patient $P_{i}$ (in a group of multiple patients) and servers $S_{j}$ (e.g., private doctors, genomic data scientists, etc.) who communicate with each other for conducting group services. DNA-based U-healthcare includes various services, namely, disease virus control, body fluid monitoring, blood-based prognostic tracking, and so on [3,40]. Taking family healthcare services as an example, multiple members $P_{i}$ in a family may request a common DNA-based healthcare service provided by $S_{j}$. The service allows the family members to obtain medical data shared among them and to know of the health status of each other conveniently. As a spiritual element, family plays an important role in promoting our health as well as in improving quality of life [41]. In case of need, a family member may also render timely assistance to doctors in observing the other members’ states of illness. Thus, it would significantly improve efficiency of long-term care or treatments and help in reducing the risk of medical incidents. To trigger the services, biological samples of $P_{i}$ (e.g., saliva) are loaded into the sequencer ${SD}_{i}$ that is inserted into $P_{i}$’s mobile device ${MD}_{i}$ in advance. Next, an onsite sequencing and data analysis process is run directly on ${SD}_{i}$; the DNA sequencing data generated is transmitted to $S_{j}$ for point-of-care services. This procedure is secured by the group session key distributed by $S_{j}$ to $P_{i}$ in the proposed protocol. Since all patients receive an identical key from $S_{j}$, $P_{i}$ is also able to share the data with other patients in the group. Thereafter, analytical results and related medical information based on the received DNA data are encrypted by the key before being sent back to a single patient or to multiple patients of the group. In the proposed architecture, these communications are carried out via the IR signal of the 6G technology. Due to its extremely high data transfer rate, 6G can offer a fully seamless experience for real-time U-healthcare services with large data sets produced by onsite mobile DNA sequencing. $P_{i}$ can enjoy the services without constraints of time and physical location. As mentioned, a dynamic healthcare solution is also introduced in the system which allows the services to be flexibly allotted by separate time-bounds based on specific requests. Furthermore, the author recommends integrating some related advances, e.g., WBAN, into the system to enhance efficiency of the overall healthcare treatment process.

Prior to starting using the above services, $P_{i}$ should register with multiple $S_{j}$ using three factors, namely, password ${PW}_{i}$, sequencing device ${SD}_{i}$, and his/her biometrics $B_{i}$, establishing a multi-server communication environment. In order to receive the group keys, $P_{i}$ uses a single set of registered credentials stored in ${SD}_{i}$ to carry out the SD-SSO that sends a login and authentication request to $S_{j}$ through a public IoLT network. In such an unreliable channel, there are possible security attacks that may induce serious consequences, e.g., violating patient privacy, destructing system architecture, or reducing reliability and quality of service, etc. Based on the author’s observation, an adversary 𝒜 may have the following capabilities to attack the proposed communication system.

- 𝒜 has full control over the open IoLT, which enables 𝒜 to intercept, insert, delete, or replay any transcripts conveyed between $P_{i}$ and $S_{j}$.
- 𝒜 may attempt to attack the past communications between $P_{i}$ and $S_{j}$ based on secret parameters or on a group session key 𝒜 somehow retrieves from the current communicated messages.
- 𝒜 may attempt to extract the secret values or registered credentials stored in a compromised ${SD}_{i}$ and use them to attack the communication.
- 𝒜 may be a privileged insider (e.g., member of a maintenance team) who can launch even more serious attacks upon a patient’s registered information obtained from ${DB}_{j}$.
- 𝒜 may also be a corrupted $P_{i}$ or $S_{j}$ that can trigger similar attacks on the communication.

### 4.2. Formal Security Model

Real-or-Random (RoR) is a well-known formal model used for analyzing success probability of an adversary in attacking cryptographic protocols [42]. In the model, suppose there are two main entities including a patient P and a server S who are communicating with each other via a public channel. Ç denotes a protocol challenger while the message communicated by P and S is denoted as m. The following queries should be executed by an adversary 𝒜 to make various attacks.

(1) *Send*(Ç, m): This query allows 𝒜 to request a message m to Ç; Ç replies to 𝒜 based upon the procedure of the proposed protocol.
(2) *Execute*(P,S): In this query, 𝒜 is allowed to eavesdrop the message m conveyed between P and S.
(3) *Reveal*(Ç): This query enables 𝒜 to retrieve a session key computed by Ç in accordance with the procedure of the protocol.
(4) *Corrupt*(P,w): In a three-factor authentication protocol, this query returns to 𝒜 password ${PW}_{i}$, parameters stored in sequencing device ${SD}_{i}$, and biometrics $B_{i}$ if w=1, w=2, and w=3, respectively.
(5) *Test*(Ç): This is a statistical test query. 𝒜 is allowed to directly request Ç for the session key; Ç probabilistically replies to 𝒜 upon the outcome of a tossed coin b.

**Definition 6.**  *Let ${Adv}_{Ç}^{IoLTHC}$ be the advantage of 𝒜 running in polynomial time in a semantically breaking security system of the proposed protocol. We have ${Adv}_{Ç}^{IoLTHC}=\left|2\mathrm{Pr}\left[b^{\prime }=b\right]-1\right|$, where IoLTHC stands for IoLT-based healthcare and $b^{\prime }$ is denoted as a guessed bit of the key.*

## 5. The Proposed Protocol

There are five procedures in the proposed protocol, including setup; user registration, login, and authentication; synchronizable key derivation; and password and biometrics change. For facilitating NDA-based U-healthcare processes, $S_{j}$ is allowed to securely distribute a common key to a group of multiple $P_{i}$. The design details are as follows.

### 5.1. Setup Phase

At first, the system selects an elliptic curve over a finite field *Fp* $Ep\left(a,b\right):y^{2}=x^{3}+ax+b(mod\;p)$ with a basic point $P_{\left(x,y\right)}$ of the order n of an additive cyclic group, where p is *k*-bit prime and n is a large number. For a neat design, the coordinates x and y of $P_{\left(x,y\right)}$ are always ignored during procedures of the protocol. $S_{m}$ chooses a secret private key ${prk}_{j}$ and computes its public key ${puk}_{j}={prk}_{j}\cdot P$.

### 5.2. Registration Phase

**Figure 3:**
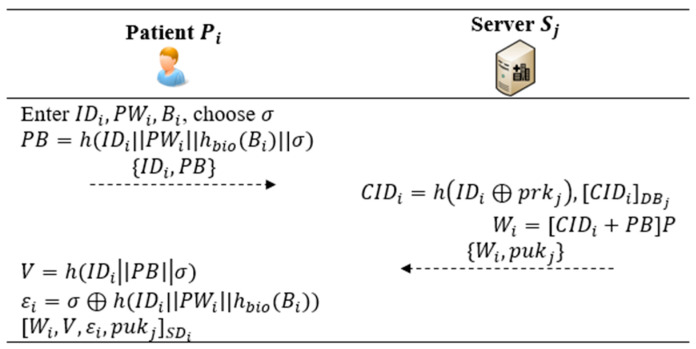
Registration procedure of the proposed protocol.

This phase is carried out via a secure channel. $P_{i}$ registers with $S_{j}$ to become a legitimate patient for using U-healthcare services. As depicted in Figure 3, $P_{i}$ and $S_{j}$ perform the following steps for this procedure.

*Step R1*: $P_{i}$ inserts ${SD}_{i}$ into ${MD}_{i}$ and selects an identity ${ID}_{i}$, a password ${PW}_{i}$, and a biometrics value $B_{i}$. $P_{i}$ selects a random number σ, and computes $PB=h({ID}_{i}|\left|{PW}_{i}\right||h_{bio}(B_{i})||\sigma )$. Next, $P_{i}$ sends $\left\{{ID}_{i},PB\right\}$ to $S_{j}$.

*Step R2*: Receiving the message $\left\{{ID}_{i},PB\right\}$, $S_{j}$ computes ${CID}_{i}=h\left({ID}_{i}⊕{prk}_{j}\right)$ and checks if ${CID}_{i}$ exists in ${DB}_{j}$, which can trace registered users for achieving patient-centered services in the U-healthcare system. Next, $S_{j}$ computes $W_{i}=[{CID}_{i}+PB]P$, stores ${CID}_{i}$ in ${DB}_{j}$, and sends $\left\{W_{i},puk\right\}$ to $P_{i}$.

*Step R3*: Upon the received $\left\{W_{i},{puk}_{j}\right\}$, $P_{i}$ computes $V=h({ID}_{i}\left|\left|PB\right|\right|\sigma )$ and $\varepsilon_{i}=\sigma ⊕h({ID}_{i}|\left|{PW}_{i}\right||h_{bio}(B_{i}))$. Finally, $P_{i}$ stores $\left\{W_{i},V,\varepsilon_{i},{puk}_{j}\right\}$ in ${SD}_{i}$ and the registration is completed. In this way, the service availability is enabled on multiple mobile devices ${MD}_{i}$.

**Remark 1:**  *Each $P_{i}$ has a unique value of ${CID}_{i}$ stored in ${DB}_{j}$. Based on ${CID}_{i}$, $S_{j}$ can easily identify $P_{i}$, refer to the past records, and focus on the particular care needs of $P_{i}$, enabling patient-centered services.*

### 5.3. Login and Authentication Phase

**Figure 4:**
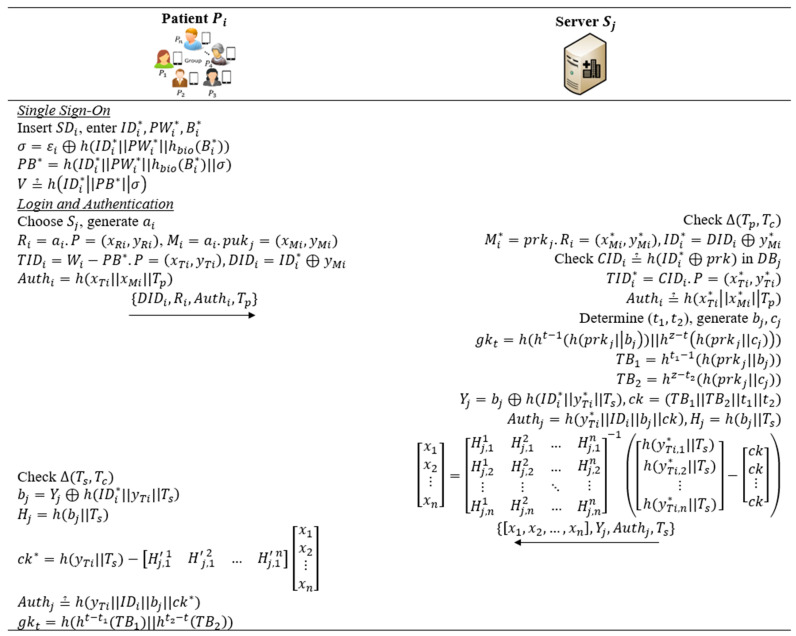
Login and authentication procedure of the proposed protocol.

This procedure is carried out via a public channel. $P_{i}$ uses their registered credentials to login to $S_{j}$. $P_{i}$ and $S_{j}$ authenticate with each other and compute a secret shared session key used for group healthcare communications. Suppose there are n patients participating in a group communication, Figure 4 presents the procedure where a session key is established.

*Step A1*: $P_{i}$ inserts ${SD}_{i}$ into ${MD}_{i}$ and enters credentials ${ID}_{i}^{*},{PW}_{i}^{*},B_{i}^{*}$. $P_{i}$ computes $\sigma_{i}=\varepsilon_{i}⊕h({ID}_{i}^{*}|\left|{PW}_{i}^{*}\right||h_{bio}(B_{i}^{*}))$ and ${PB}^{*}=h({ID}_{i}^{*}|\left|{PW}_{i}^{*}\right||h_{bio}(B_{i}^{*})||\sigma )$. The check $V≟h({ID}_{i}^{*}||{PB}^{*}||\sigma ))$ is performed. If the check holds, the SD-SSO is completed and $P_{i}$ is allowed to select a server $S_{j}$ in the interface of an app installed in ${MD}_{i}$ for enjoying a specific service. To this end, $P_{i}$ chooses a random number $a_{s}$ and a timestamp $T_{p}$, then computes $R_{i}=a_{i}\cdot P=(x_{Ri},y_{Ri})$, $M_{i}=a_{i}\cdot {puk}_{j}=(x_{Mi},y_{Mi})$, ${TID}_{i}=W_{i}-{PB}^{*}\cdot P=\left(x_{Ti},y_{Ti}\right)$, ${DID}_{i}={ID}_{i}^{*}⊕y_{Mi}$, and ${Auth}_{i}=h(x_{Ti}||x_{Mi}||T_{p})$. $P_{i}$ conveys message $\left\{{DID}_{i},R_{i},{Auth}_{i},T_{p}\right\}$ to $S_{j}$ for the purposed of login.

*Step A2*: Upon receiving the message $\left\{{DID}_{i},R_{i},{Auth}_{i},T_{p}\right\}$, $S_{j}$ checks the timestamp $T_{p}$ and computes $M_{i}^{*}={prk}_{j}\cdot R_{i}=(x_{Mi}^{*},y_{Mi}^{*})$ and ${ID}_{i}^{*}={DID}_{i}⊕y_{Mi}^{*}$. $S_{j}$ checks ${CID}_{i}≟h({ID}_{i}^{*}⊕{prk}_{j})$ in its database and checks ${Auth}_{i}≟h(x_{Ti}^{*}\left|\left|x_{Mi}^{*}\right|\right|T_{p})$ for confirming the legitimacy of $P_{i}$. Next, $S_{j}$ determines a time bound $\left(t_{1},t_{2}\right)$, chooses two random numbers $b_{j},c_{j}$, and computes a group dynamic key at a time point t by ${gk}_{t}=h(h^{t-1}(h({prk}_{j}|\left|b_{j}\right))||h^{z-t}\left(h({prk}_{j}||c_{j})\right))$. $S_{j}$ computes ${TB}_{1}=h^{t_{1}-1}(h({prk}_{j}||b_{j}))$, ${TB}_{2}=h^{z-t_{2}}(h({prk}_{j}||c_{j}))$, $Y_{j}=b_{j}⊕h({ID}_{i}^{*}|\left|y_{Ti}^{*}\right||T_{s})$, $ck=({TB}_{1}||{TB}_{2}||t_{1}||t_{2})$, and $H_{j}=h(b_{j}||T_{s})$. The value ck is masked by generating multiple $\left[\begin{array}{c} x_{1}\\ x_{2}\\ \begin{array}{c} \vdots \\ x_{n} \end{array} \end{array}\right]={\left[\begin{array}{ccc} \begin{array}{c} H_{j,1}^{1}\\ H_{j,2}^{1}\\ \vdots \end{array} & \begin{array}{cc} H_{j,1}^{2} & \ldots \\ H_{j,2}^{2} & \ldots \\ \vdots & \ddots \end{array} & \begin{array}{c} H_{j,1}^{n}\\ H_{j,2}^{n}\\ \vdots \end{array}\\ H_{j,n}^{1} & \begin{array}{cc} H_{j,n}^{2} & \ldots \end{array} & H_{j,n}^{n} \end{array}\right]}^{-1}\left(\left[\begin{array}{c} \begin{array}{c} h(y_{Ti,1}^{*}||T_{s})\\ h(y_{Ti,2}^{*}||T_{s}) \end{array}\\ \vdots \\ h(y_{Ti,n}^{*}||T_{s}) \end{array}\right]-\left[\begin{array}{c} ck\\ ck\\ \begin{array}{c} \vdots \\ ck \end{array} \end{array}\right]\right)$. $S_{j}$ conveys a message $\left\{\left[x_{1},x_{2},\ldots ,x_{n}\right],Y_{j},{Auth}_{j},T_{s}\right\}$ to $P_{i}$.

*Step A3*: Upon receiving the above message, $P_{i}$ checks the timestamp $T_{s}$ and computes $b_{j}=Y_{j}⊕h({ID}_{i}^{*}||y_{Ti}||T_{s}$, $H_{j}=h(b_{j}||T_{s})$, and ${ck}^{*}=h(y_{Ti}||T_{s})-\left[\begin{array}{cc} H_{j,1}^{\prime 1} & \begin{array}{ccc} H_{j,1}^{\prime 2} & \ldots & H_{j,1}^{\prime n} \end{array} \end{array}\right]\left[\begin{array}{c} x_{1}\\ x_{2}\\ \begin{array}{c} \vdots \\ x_{n} \end{array} \end{array}\right]$. Next, $S_{j}$ checks ${Auth}_{j}≟h(y_{Ti}||{ID}_{i}||b_{j}||{ck}^{*})$. If the check holds, the value ${ck}^{*}=({TB}_{1}||{TB}_{2}||t_{1}||t_{2})$ is successfully verified. $S_{j}$ computes the dynamic group key at the time point t by ${gk}_{t}=h(h^{t-t_{1}}({TB}_{1})||h^{t_{2}-t}\left({TB}_{2}\right))$. In this way, all members $P_{i}$ in a group of n patients have received the same key ${gk}_{t}$ for U-healthcare communications.

**Remark 2:**  *The design allows the time bound $\left(t_{1},t_{2}\right)$ to be flexibly changed without having to renew the registration. $P_{i}$ would be notified of the updated time bound through the app’s notification during the communication session or through some channel (e.g., email) before the communication gets started.*

**Remark 3:**  *Upon specific requests, $P_{i}$ and $S_{j}$ are allowed to compute multiple group keys at different time point* *s t by ${gk}_{t}=h(h^{t-1}(h({prk}_{j}|\left|b_{j}\right))||h^{z-t}\left(h({prk}_{j}||c_{j})\right))$ and by ${gk}_{t}=h(h^{t-t_{1}}({TB}_{1})||h^{t_{2}-t}\left({TB}_{2}\right))$, respectively. The key ${gk}_{t}$ is used as a symmetric encryption key to protect communications between $S_{j}$ and multiple $P_{i}$, and between $P_{i}$ and $P_{i}$.*

### 5.4. Synchronizable Key-Derivation Phase

**Figure 5:**
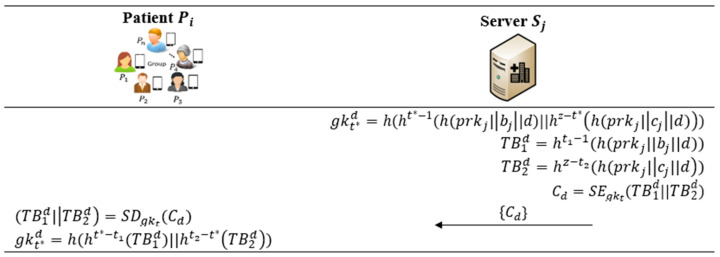
Synchronizable key-derivation procedure of the proposed protocol.

In this procedure, $P_{i}$ and $S_{j}$ are allowed to quickly compute a new group key to enhance security and to address desynchronization problems in patient–patient communications or in patient–server communications. For example, $S_{j}$ distributes a key ${gk}_{8}$ at 8:00 a.m. to the group; then, $S_{j}$ uses this key for encrypting the data; if a patient $P_{i}$ joins the communication at 9:00 a.m. and obtains the key ${gk}_{9}$, $P_{i}$ is not able to decrypt the data encrypted using ${gk}_{8}$. It is likely that multiple patients would be in this situation or that some similar situations happen at the same time. This causes a serious communicational desynchronization in the system, since multiple keys would be generated at different time points for a single service. To this end, two values ${TB}_{1}$ and ${TB}_{2}$ should be renewed in order to reset the communication with a new common key computed without having to repeat the many steps of the previous procedure. Figure 5 describes specific steps performed in this phase.

*Step D1*: $S_{j}$ generates a number d, which can be regarded as the number of key derivations. Upon a time point $t^{*}$, $S_{j}$ computes a new group key ${gk}_{t^{*}}^{d}=h(h^{t^{*}-1}(h({prk}_{j}|\left|b_{j}\right||d)||h^{z-t^{*}}(h({prk}_{j}|\left|c_{j}\right||d)))$ and two new values ${TB}_{1}^{d}=h^{t_{1}-1}(h({prk}_{j}||b_{j}||d))$ and ${TB}_{2}^{d}=h^{z-t_{2}}(h({prk}_{j}|\left|c_{j}||d\right))$. $S_{j}$ generates a symmetric ciphertext $C_{d}={SE}_{{gk}_{t}}({TB}_{1}^{d}||{TB}_{2}^{d})$ using previous key ${gk}_{t}$, and conveys $\left\{C_{d}\right\}$ to $P_{i}$.

*Step D2*: Upon receiving the message, $P_{i}$ decrypts $C_{d}$ and obtains ${TB}_{1}^{d},{TB}_{2}^{d}$. Finally, $P_{i}$ computes the new group key by ${gk}_{t^{*}}^{d}=h(h^{t^{*}-t_{1}}({TB}_{1}^{d})||h^{t_{2}-t^{*}}\left({TB}_{2}^{d}\right))$ at the time point $t^{*}$. In this way, the key ${gk}_{t}$ computed in the previous phase (Section 5.3) is changed to the key ${gk}_{t^{*}}^{d}$ for resolving possible desynchronization issues of similar communications.

**Remark 4:**  The time point t and the time point $t^{*}$ may or may not be identical based on the time allocation of specific services.

### 5.5. Password and Biometrics Change Phase

**Figure 6:**
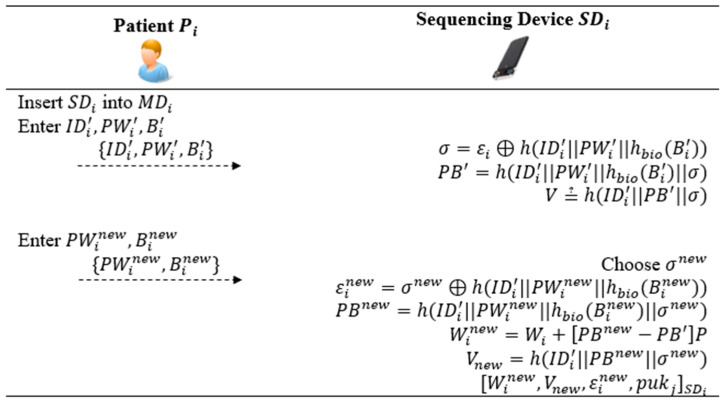
Password and biometrics change procedure of the proposed protocol.

This procedure allows $P_{i}$ to change their password and biometrics to enhance security. As shown in Figure 6, $P_{i}$ and ${SD}_{i}$ perform the following steps for updating these credentials.

*Step C1*: $P_{i}$ inserts ${SD}_{i}$ and enters ${ID}_{i}^{\prime },{PW}_{i}^{\prime },B_{i}^{\prime }$. ${MD}_{i}$ computes $\sigma =\varepsilon_{i}⊕h({ID}_{i}^{\prime }|\left|{PW}_{i}^{\prime }\right||h_{bio}(B_{i}^{\prime }))$ and ${PB}^{\prime }=h({ID}_{i}^{\prime }|\left|{PW}_{i}^{\prime }\right||h_{bio}(B_{i}^{\prime })||\sigma )$. It checks $V≟h({ID}_{i}^{\prime }||{PB}^{\prime }||\sigma )$. If the check holds, $P_{i}$ is requested to enter new password ${PW}_{i}^{new}$ and new biometrics $B_{i}^{new}$.

*Step C2*: Receiving ${PW}_{i}^{new},B_{i}^{new}$ from $P_{i}$, ${SD}_{i}$ chooses a new $\sigma^{new}$ and computes $\varepsilon_{i}^{new}=\sigma^{new}⊕h({ID}_{i}^{\prime }|\left|{PW}_{i}^{new}\right||h_{bio}(B_{i}^{new}))$*,* ${PB}^{new}=h({ID}_{i}^{\prime }|\left|{PW}_{i}^{new}\right||h_{bio}(B_{i}^{new})||\sigma^{new})$*,* $W_{i}^{new}=W_{i}+\left[{PB}^{new}-PB\right]P$, and $V_{new}=h({ID}_{i}^{\prime }||{PB}^{new}||\sigma^{new})$. ${MD}_{i}$ replaces $W_{i},V,\varepsilon_{i}$ with $W_{i}^{new},V_{new},\varepsilon_{i}^{new}$ in ${SD}_{i}$.

## 6. Security Certificate

In this section, the author provides the security certificate of the proposed protocol. An informal discussion, a logical analysis using BAN logic, and a formal mathematical proof using the RoR model are included for security evaluation as follows.

### 6.1. Sematic Security Discussion

In this subsection, the prevention of various well-known attacks in the protocol is presented in a detailed manner. The author also discusses multiple functionalities and security features achieved by the proposed work.

(1) *Replay attacks*: Suppose the message $\left\{{DID}_{i},R_{i},{Auth}_{i},T_{p}\right\}$ is intercepted by 𝒜 and it is resent to $S_{m}$ to launch a replay attack in the next session. However, timestamp $T_{p}$ in the protocol is employed to check if the message is resent. Moreover, when receiving the message $\left\{\left[x_{1},x_{2},\ldots ,x_{n}\right],Y_{j},{Auth}_{j},T_{s}\right\}$, 𝒜 will also fail to compromise the key ${gk}_{t}$ since 𝒜 does not know of ${ID}_{i},y_{Ti}$ for retrieving the number $b_{j}$. Therefore, the replay attack is prevented in the proposed protocol.
(2) *MITM attacks*: On the received message $\left\{{DID}_{i},R_{i},{Auth}_{i},T_{p}\right\}$, 𝒜 may insert forged parameters and generate a candidate login message. 𝒜 aims to act as a middle man to compromise the conveyed messages without being noticed by $P_{i}$ and $S_{j}$. However, without the private key ${prk}_{j}$, 𝒜 is not able to compute sufficient parameters for the verifications on ${CID}_{i}$ and ${Auth}_{i}$. Similarly, without $y_{Ti}$ and ${ID}_{i}$, 𝒜 can also not compute a valid message $\left\{\left[x_{1},x_{2},\ldots ,x_{n}\right],Y_{j},{Auth}_{j},T_{s}\right\}$ for the check on ${Auth}_{j}$ on the patient side. As a result, the protocol is free from MITM attacks.
(3) *Password and biometrics guessing attacks:* At first, 𝒜 may attempt to directly enter a candidate password for logging to the system. However, the login request will be immediately rejected by ${SC}_{i}$ upon the check $V≟h({ID}_{i}^{*}||{PB}^{*}||\sigma )$. Suppose the hash value PB is somehow known to 𝒜, then 𝒜 attempts to guess ${PW}_{i}$ based on PB. Other than ${PW}_{i}$, the values ${ID}_{i},{PW}_{i},h_{bio}\left(B_{i}\right),\sigma$ are also included in the function generating PB. Therefore, it is extremely hard (with a negligible success probability) for 𝒜 to guess the correct ${PW}_{i}$ by computing candidate hashes and comparing them with the original PB. Using similar arguments, the biometrics $B_{i}$ is also completely protected during the communication process. Moreover, my work provides password and biometrics update functions that further assure the security of ${PW}_{i}$ and $B_{i}$. Therefore, a robust three-factor authentication mechanism is achieved in the proposed protocol.
(4) *Impersonation attacks:* Suppose the identity ${ID}_{i}$ is somehow disclosed, then 𝒜 obtains and uses it to generate a fake login message for impersonating $P_{i}$. However, it is not possible for 𝒜 to launch this impersonation attack without ${PW}_{i},B_{i}$ since the protocol can resist password and biometrics guessing attacks, as stated above. Moreover, without the knowledge of $y_{Ti}$, 𝒜 can also not retrieve $b_{j}$ for further steps upon the known ${ID}_{i}$. Thus, impersonation attacks are resisted in the proposed protocol.
(5) *Lost/stolen sequencer attacks:* Suppose 𝒜 has somehow stolen the sequencer ${SD}_{i}$; then, 𝒜 retrieved all stored parameters. However, the important credentials ${ID}_{i},{PW}_{i},B_{i}$ are not stored in ${SD}_{i}$ directly. Obtaining the parameters $W_{i},V,\varepsilon_{i},{puk}_{j}$ inside ${SD}_{i}$ is not sufficient for passing the verification $V≟h({ID}_{i}^{*}||{PB}^{*}||\sigma )$ and for generating a valid login request message $\left\{{DID}_{i},R_{i},{Auth}_{i},T_{p}\right\}$. Thus, my protocol is robust against lost/stolen sequencer attacks.
(6) *Desynchronization attacks:* Two acknowledgement values ${Auth}_{i}$ and ${Auth}_{j}$ generated by $P_{i}$ and $S_{j}$, respectively, are used for assuring a robust mutual authentication in the proposed protocol. ${Auth}_{i}$ and ${Auth}_{j}$ are deleted after the login and authentication procedure session is completed. In addition, after each synchronizable key-derivation procedure finishes, $P_{i}$ and $S_{j}$ do not update or store any redundant parameters used for the next communication sessions. Hence, desynchronization problems and related attacks are prevented in my work.
(7) *Privileged insider attacks:* Suppose there is a privileged insider 𝒜 who can monitor data transmission during the registration and capture message $\left\{{ID}_{i},PB\right\}$. Upon the reception of ${ID}_{i}$, it is not possible for 𝒜 to compromise the communication due to the stated resistance to impersonation attacks. Using the value PB, 𝒜 is also not able to compute a correct ${TID}_{i}$ for the attack on ${Auth}_{i}$ without $W_{i}$ stored in the smart card. In another scenario, even if 𝒜 somehow obtains ${CID}_{i}$ in the database, 𝒜 still cannot pass the server verification without ${ID}_{i}$. Thus, the protocol can resist privileged insider attacks.
(8) *DoS attacks:* For analysis of DoS attacks, the author discusses some possible threats that may affect communication performance of the protocol. In the login phase, the system verifies $P_{i}$ by $V≟h({ID}_{i}^{*}||{PB}^{*}||\sigma )$ upon the newly input credentials ${ID}_{i}^{*},{PW}_{i}^{*},B_{i}^{*}$. If the check is not successful, the session will be immediately terminated. Hence, it is not possible for 𝒜 is not able to flood the login and authentication procedure using multiple subsequent steps. On the other hand, upon the received message from $P_{i}$, $S_{j}$ only operates two minor computations $M_{i}^{*}={prk}_{j}\cdot R_{i}$ and ${ID}_{i}^{*}={DID}_{i}⊕y_{Mi}^{*}$ before the check ${CID}_{i}≟h({ID}_{i}^{*}⊕{prk}_{j})$ is made. Retransmitting massive messages $\left\{{DID}_{i},R_{i},{Auth}_{i},T_{p}\right\}$ to $S_{j}$ for disrupting its services would not be an efficient attack due to the redundant resources of $S_{j}$. Moreover, the communication will also be terminated once the check $∆(T_{p},T_{c})$ does not hold in the beginning. Therefore, DoS attacks are prevented in the protocol.
(9) *Robust mutual authentication*: In the proposed communication, $P_{i}$ should be authenticated as a legitimate patient for preventing patients’ identities and possibly costly services from being compromised. Upon receiving the login request $\left\{{DID}_{i},R_{i},{Auth}_{i},T_{p}\right\}$ from $P_{i}$, using the private key, $S_{j}$ computes $M_{i}^{*}$ and retrieves ${ID}_{i}^{*},{CID}_{i},{DID}_{i}$. These parameters are used for the verification ${Auth}_{i}≟h(x_{Ti}^{*}\left|\left|x_{Mi}^{*}\right|\right|T_{p})$ that confirms the legitimacy of the patient $P_{i}$. On the other hand, based on the message $\left\{\left[x_{1},x_{2},\ldots ,x_{n}\right],Y_{j},{Auth}_{j},T_{s}\right\}$, $P_{i}$ retrieves the number $b_{j}$ to compute $H_{j},{ck}^{*}$. These parameters are used for the check ${Auth}_{j}≟h(y_{Ti}||{ID}_{i}||b_{j}||{ck}^{*})$ of the acknowledgement that confirms legitimacy of the server $S_{j}$ and assures true service provision. If one of the above checks fails, the session will be terminated and the session key will not be established successfully. Hence, a robust mutual authentication is achieved in the proposed protocol.
(10) *Patient anonymity and untraceability*: The identity ${ID}_{i}$ is hidden in the parameter ${DID}_{i}$ of the login message $\left\{{DID}_{i},R_{i},{Auth}_{i},T_{p}\right\}$ requested by $P_{i}$. Also, the message $\left\{\left[x_{1},x_{2},\ldots ,x_{n}\right],Y_{j},{Auth}_{j},T_{s}\right\}$ sent by $S_{j}$ does not reveal ${ID}_{i}$ to the public. Therefore, the anonymity of ${ID}_{i}$ is guaranteed during the login and authentication process. The parameters contained in $\left\{{DID}_{i},R_{i},{Auth}_{i},T_{p}\right\}$ and $\left\{\left[x_{1},x_{2},\ldots ,x_{n}\right],Y_{j},{Auth}_{j},T_{s}\right\}$ in respective communication sessions are totally not identical since different random numbers and timestamps are used for the computations. Therefore, 𝒜 is not able to identify any two login messages sent by the same patient $P_{i}$. Hence, the proposed protocol achieves patient anonymity and patient untraceability.
(11) *Message unlinkability*: When linking the parameters of all messages $\left\{{DID}_{i},R_{i},{Auth}_{i},T_{p},\left[x_{1},x_{2},\ldots ,x_{n}\right],Y_{j},{Auth}_{j},T_{s}\right\}$ to each other, there are not any fixed values found. It means that it will not allow 𝒜 to trace $P_{i}$ for the purpose of guessing $P_{i}$’s identity. Thus, a message unlinkability feature is achieved in the proposed protocol.
(12) *Perfect forward secrecy*: Suppose some sensitive data, secret parameters, or even a session key established in the current session are somehow revealed to 𝒜. Upon receiving these vales, 𝒜 attempts to attack the past communications. However, it is not possible for 𝒜 to launch the attack since the values are completely not identical in different communication sessions due to the inclusion of random numbers and timestamp values in the computations. For instance, 𝒜 cannot use the currency key ${gk}_{t}^{current}=h(h^{t-t_{1}}({TB}_{1})||h^{t_{2}-t}\left({TB}_{2}\right))$ to compromise the message encrypted using a key ${gk}_{t}^{past}$ established in the past session. If the long-term private key ${prk}_{j}$ of $S_{j}$ is compromised, the secrecy of ${gk}_{t}^{past}$ is also not affected, because there are no associated parameters between them. Hence, a perfect forward secrecy is achieved in my protocol.
(13) *Perfect backward secrecy (known-key security)*: With similar arguments, the protocol is proven not to be vulnerable to a known-key attack, since compromise of the past key ${gk}_{t}^{past}$ does not allow either a passive 𝒜 to compromise the future key ${gk}_{t}^{future}$ or impersonation by an active 𝒜 in the future.

### 6.2. Logical Analysis Using BAN logic

In this subsection, the well-known BAN logic [43] is employed to further provide a logical analysis on the mutual authentication between $P_{i}$ and $S_{j}$. Some rules and analytical logics in the tool are defined in advance. Next, the analysis demonstrates that $P_{i}$ and $S_{j}$ believe the key ${gk}_{t}$ is a secret value shared between them only. Some notations used for the analysis are provided in Table 2.

In accordance with the principle of BAN logic and operation rules in my proposed protocol, the mutual authentication proof should satisfy the following four goals. In the protocol, the value ck is utilized by $S_{j}$ to distribute ${TB}_{1}$ and ${TB}_{2}$ to $P_{i}$ for computing the group key ${gk}_{t}$. Therefore, authenticity of both ck and ${gk}_{t}$ should be proven, which can guarantee a completely authenticated key shared between the entities.

***Goal* 1:** $S_{j}$ |≡ $\left(P_{i}\overset{{gk}_{t}}{↔}S_{j}\right)$. $S_{j}$ believes that the key ${gk}_{t}$ computed is a secret value shared between $P_{i}$ and $S_{j}$. (**G1**)

***Goal* 2:** $S_{j}$ |≡ $\left(P_{i}\overset{ck}{↔}S_{j}\right)$. $S_{j}$ believes that the key ck computed is a secret value shared between $P_{i}$ and $S_{j}$. (**G2**)

***Goal* 3:** $P_{i}$ |≡ $\left(P_{i}\overset{ck}{↔}S_{j}\right)$. $P_{i}$ believes that the key ck computed is a secret value shared between $P_{i}$ and $S_{j}$. (**G3**)

***Goal* 4:** $P_{i}$ |≡ $\left(P_{i}\overset{{gk}_{t}}{↔}S_{j}\right)$. $P_{i}$ believes that the key ${gk}_{t}$ computed is a secret value shared between $P_{i}$ and $S_{j}$. (**G4**)

Two messages communicated in the login and authentication procedure of the protocol are included in the authentication proof.

*Message 1*: $P_{i}\to S_{j}:({ID}_{i}^{*}⊕y_{Mi},x_{Ri},y_{Ri},h(x_{Ti}||x_{Mi}||T_{p}),T_{p})$

*Message 2*: $S_{j}\to P_{i}:(\left[x_{1},x_{2},\ldots ,x_{n}\right],b_{j}⊕h({ID}_{i}^{*}|\left|y_{Ti}^{*}\right||T_{s}),h(y_{Ti}^{*}||{ID}_{i}||b_{j}|\left|ck\right),T_{s})$

Some logical rules of the tool used in the proof are provided as follows.

- Seeing rule (R1): $\frac{X\overset{K}{\Leftrightarrow }Y,Y⊲{\left\langle A\right\rangle }_{K}}{X\left|\equiv Y\right|\sim A}$;
- Interpretation rule (R2): $\frac{X\left|\equiv Y\right|\sim (A,B)}{X\left|\equiv Y\right|\sim A}$;
- Freshness rule (R3): $\frac{X|\equiv \#(A)}{X|\equiv \#(A,B)}$;
- Verification rule (R4): $\frac{X|\equiv \#\left(A\right),X|\equiv Y|\sim A}{X|\equiv Y|\equiv A}$;
- Jurisdiction rule (R5): $\frac{X|\equiv Y⟹A,X|\equiv Y|\equiv A}{X|\equiv A}$;
- Belief rule (R6): $\frac{X|\equiv (A,B)}{X|\equiv A}$.

Along with the rules, the following assumptions are also used in the analysis.

- Assumption 1 (A1): $S_{j}$ |≡ $P_{i}\overset{K_{ij}}{\Leftrightarrow }S_{j}$;
- Assumption 2 (A2): $S_{j}$ |≡ $\#(T_{p})$;
- Assumption 3 (A3): $S_{j}|\equiv P_{i}⟹(x_{Ti},x_{Mi},T_{p})$;
- Assumption 4 (A4): $S_{j}⟹(t_{1})$;
- Assumption 5 (A5): $S_{j}⟹(t_{2})$;
- Assumption 6 (A6): $S_{j}⟹(b_{j})$;
- Assumption 7 (A7): $S_{j}⟹(c_{j})$;
- Assumption 8 (A8): $S_{j}⟹({prk}_{j})$;
- Assumption 9 (A9): $P_{i}$ |≡ $\#(T_{s})$;
- Assumption 10 (A10): $P_{i}|\equiv S_{j}⟹(\left[x_{1},x_{2},\ldots ,x_{n}\right],b_{j},y_{Ti},T_{s})$;
- Assumption 11 (A11): $P_{i}⟹({ID}_{i})$.

In this way, an idealized form of the communicated messages is described as follows.

*Message 1*: $P_{i}\to S_{j}:({\left\langle ID_{i},y_{Mi}\right\rangle }_{K_{ij}},x_{Ri},y_{Ri},{\left\langle x_{Ti},x_{Mi},T_{p}\right\rangle }_{K_{ij}},T_{p})$

*Message 2*: $S_{j}\to P_{i}:([x_{1},x_{2},\ldots ,x_{n}],{\left\langle b_{j},ID_{i},y_{Ti},T_{s}\right\rangle }_{K_{ij}},{\left\langle y_{Ti},ID_{i},b_{j},ck\right\rangle }_{K_{ij}},T_{s})$

Based on the specified rules, assumptions, and procedure of the protocol, the logical analysis of mutual authentication between $P_{i}$ and $S_{j}$ in the proposed protocol is described by the following steps.

- ${Step}_{1}$: Based on the *Message* 1, we have $S_{j}⊲({\left\langle ID_{i},y_{Mi}\right\rangle }_{K_{ij}},x_{Ri},y_{Ri},{\left\langle x_{Ti},x_{Mi},T_{p}\right\rangle }_{K_{ij}},T_{p})$.
- ${Step}_{2}$: Using A1 and R1, we have $S_{j}$ |≡ $P_{i}$ |~ $\left({ID}_{i},y_{Mi},x_{Ri},y_{Ri},x_{Ti},x_{Mi},T_{p}\right)$.
- ${Step}_{3}$: According to R2, we obtain $S_{j}$ |≡ $P_{i}$ |~ $\left(x_{Ti},x_{Mi},T_{p}\right)$.
- ${Step}_{4}$: Using R3 and A2, we have $S_{j}$ |≡ #$\left(x_{Ti},x_{Mi},T_{p}\right)$.
- ${Step}_{5}$: Based on R4, ${Step}_{3}$, and ${Step}_{4}$, we obtain $S_{j}$ |≡ $P_{i}$ |≡ $\left(x_{Ti},x_{Mi},T_{p}\right)$.
- ${Step}_{6}$: According to R5, A3, and ${Step}_{5}$, we obtain $S_{j}$ |≡ $\left(x_{Ti},x_{Mi},T_{p}\right)$.
- ${Step}_{7}$: Based on R6 and ${Step}_{6}$, we obtain $S_{j}$ |≡ $x_{Ti}$, $S_{j}$ |≡ $x_{Mi}$, and $S_{j}$ |≡ $T_{p}$.
- ${Step}_{8}$: Due to ${Step}_{7}$, and ${Auth}_{i}=h(x_{Ti}||x_{Mi}||T_{p})$, we obtain $S_{m}$ |≡ ${Auth}_{i}$.
- ${Step}_{9}$: Based on ${Step}_{8}$, A4, A5, A6, A7, A8, and ${gk}_{t}=h(h^{t-1}(h({prk}_{j}|\left|b_{j}\right))||h^{z-t}\left(h({prk}_{j}||c_{j})\right))$, we can obtain $S_{j}$ |≡ $\left(P_{i}\overset{{gk}_{t}}{↔}S_{j}\right)$ (**G1** achieved).
- ${Step}_{10}$: Based on ${Step}_{8}$, A4, A5, A6, A7, A8, and $ck=(h^{t_{1}-1}(h({prk}_{j}||b_{j}))||h^{z-t_{2}}(h({prk}_{j}||c_{j}))||t_{1}||t_{2})$, we can obtain $S_{j}$ |≡ $\left(P_{i}\overset{ck}{↔}S_{j}\right)$ (**G2** achieved).
- ${Step}_{11}$: According to the *Message* 2, we have $P_{i}⊲([x_{1},x_{2},\ldots ,x_{n}],{\left\langle b_{j},ID_{i},y_{Ti},T_{s}\right\rangle }_{K_{ij}},{\left\langle y_{Ti},ID_{i},b_{j},ck\right\rangle }_{K_{ij}},T_{s})$
- ${Step}_{12}$: In accordance with R1 and A1, we obtain $P_{i}$ |≡ $S_{m}$ |~ $\left(\left[x_{1},x_{2},\ldots ,x_{n}\right],b_{j},{ID}_{i},y_{Ti},ck,T_{s}\right)$.
- ${Step}_{13}$: Based upon R2, we can obtain $P_{i}|\equiv S_{m}|\sim (\left[x_{1},x_{2},\ldots ,x_{n}\right],b_{j},y_{Ti},T_{s})$.
- ${Step}_{14}$: Using R3 and A9, we have $P_{i}|\equiv \#(\left[x_{1},x_{2},\ldots ,x_{n}\right],b_{j},y_{Ti},T_{s})$.
- ${Step}_{15}$: Based on R4, ${Step}_{13}$ and ${Step}_{14}$, we obtain $P_{i}$ |≡ $S_{j}$ |≡ $\left(\left[x_{1},x_{2},\ldots ,x_{n}\right],b_{j},y_{Ti},T_{s}\right)$.
- ${Step}_{16}$: According to R5, A10, and ${Step}_{15}$, we obtain $P_{i}$ |≡ $\left(\left[x_{1},x_{2},\ldots ,x_{n}\right],b_{j},y_{Ti},T_{s}\right)$.
- ${Step}_{17}$: Based on R6 and ${Step}_{16}$, we obtain $P_{i}$ |≡ $\left[x_{1},x_{2},\ldots ,x_{n}\right]$, $P_{i}$ |≡ $b_{j}$, $P_{i}$ |≡ $y_{Ti}$, and $P_{i}$ |≡ $T_{s}$.
- ${Step}_{18}$: In accordance with ${Step}_{17}$, while $H_{j}=h(b_{j}||T_{s})$ and $ck=h(y_{Ti}||T_{s})-\left[\begin{array}{cc} H_{j,1}^{\prime 1} & \begin{array}{ccc} H_{j,1}^{\prime 2} & \ldots & H_{j,1}^{\prime n} \end{array} \end{array}\right]\left[\begin{array}{c} x_{1}\\ x_{2}\\ \begin{array}{c} \vdots \\ x_{n} \end{array} \end{array}\right]$, we can obtain $P_{i}$ |≡ $\left(P_{i}\overset{ck}{↔}S_{j}\right)$ (**G3** achieved).
- ${Step}_{19}$: Due to ${Step}_{17}$, ${Step}_{18}$, A11, and ${Auth}_{j}=h(y_{Ti}||{ID}_{i}||b_{j}|\left|ck\right)$, we obtain $P_{i}$ |≡ ${Auth}_{j}$.
- ${Step}_{20}$: Based on ${Step}_{18}$, ${Step}_{19}$, $ck=({TB}_{1}||{TB}_{2}||t_{1}||t_{2})$, and ${gk}_{t}=h(h^{t-t_{1}}({TB}_{1})||h^{t_{2}-t}\left({TB}_{2}\right))$, we obtain $P_{i}$ |≡ $\left(P_{i}\overset{{gk}_{t}}{↔}S_{j}\right)$ (**G4** achieved).

In this way, the proposed protocol achieves all goals—**G1**, **G2**, **G3**, and **G4**. Therefore, it proves that $P_{i}$ and $S_{j}$ can mutually authenticate each other and ${gk}_{t}$ is an authenticated key shared between them.

### 6.3. Formal Security Proof with RoR Model

Formal security proof of the proposed protocol is provided using the widely-accepted ROR model. Based on mathematical principles, its idea is to analyze the success probability of 𝒜 in attacking the protocol. The goal is to demonstrate that this probability is a negligible advantage, assuring the sematic security of the approach. Various games are included in the analysis where 𝒜 makes multiple attack queries discussed in Section 4.2 with an increased success probability. Notations used in the proof are described in Table 3.

**Definition 7.**  *When Ç receives the last communicated message in the protocol, Ç goes to an Accept state. All messages $m_{1}=\{{DID}_{i},R_{i},{Auth}_{i},T_{p}\}$ and $m_{2}=\{\left[x_{1},x_{2},\ldots ,x_{n}\right],Y_{j},{Auth}_{j},T_{s}\}$ are orderly concatenated, forming a session with an identification “s_id”.*

**Definition 8.**  $P_{i}^{T_{c}}$ and ${S_{j}}^{T_{c}^{*}}$ are defined to be partnered if $P_{i}^{T_{c}}$ and ${S_{j}}^{T_{c}^{*}}$ simultaneously meet the following conditions: (1) $P_{i}^{T_{c}}$ and ${S_{j}}^{T_{c}^{*}}$ are in an Accept state; (2) $P_{i}^{T_{c}}$ and ${S_{j}}^{T_{c}^{*}}$ mutually authenticate each other in the same session s_id; and (3) $P_{i}^{T_{c}}$ and ${S_{j}}^{T_{c}^{*}}$ are mutually a partner of each other. $P_{i}^{T_{c}}$ and ${S_{j}}^{T_{c}^{*}}$ are called “partners”.

**Definition 9.**  *Ç is defined to be fresh if Ç simultaneously meets the following conditions: (1) Ç is in an accepted state; (2) Reveal(Ç) queries have never been submitted; and (3) less than three Corrupt $\left(P_{i},n\right)$ queries have been submitted. This is called the “freshness” rule.*

**Definition 10.**  *${Adv}_{𝒜}^{ECDLP}(t_{𝒜})$ is denoted as the advantage of 𝒜 in breaking the ECDLP assumption within an execution time $t_{𝒜}$. Because the assumption holds, ${Adv}_{𝒜}^{ECDLP}(t_{𝒜})$ is a negligible probability.*

**Definition 11.**  *${Adv}_{𝒜}^{ECCDHP}(t_{𝒜})$ is denoted as the advantage of 𝒜 in breaking the ECCDHP assumption within an execution time $t_{𝒜}$. Also, ${Adv}_{𝒜}^{ECCDHP}(t_{𝒜})$ is a negligible probability since the assumption holds.*

**Definition 12.**  *${Adv}_{𝒜}^{ECFP}(t_{𝒜})$ is denoted as the advantage of 𝒜 in breaking the ECFP assumption within an execution time $t_{𝒜}$. Similarly, ${Adv}_{𝒜}^{ECFP}(t_{𝒜})$ is a negligible probability as the assumption holds.*

**Theorem 1.**  *${Adv}_{Ç}^{IoLTHC}$ can be calculated in the following equation.*

(1) $${Adv}_{Ç}^{IoLTHC}\leq \frac{{\left(q_{s}+q_{e}\right)}^{3}+6q_{s}}{2^{L_{r}}}+\frac{{q_{h}}^{2}+20q_{h}}{2^{L_{h}}}+2max\left\{\left(C^{\prime }\cdot q_{s}^{s^{\prime }}\right),q_{s}\left(\frac{1}{2^{l_{bio}}},\varepsilon_{bio}\right)\right\}\;+4q_{h}\left(q_{s}+q_{e}+1\right){Adv}_{𝒜}^{ECDLP}\left(t_{𝒜}\right)+2q_{h}(q_{s}+q_{e}+1){Adv}_{𝒜}^{ECCDHP}(t_{𝒜})\;+2q_{h}(q_{s}+q_{e}+1){Adv}_{𝒜}^{ECFP}(t_{𝒜})$$

*Since Equation (1) is obviously a negligible probability, the proposed protocol is semantically secure*.

**Proof.**  The author considers six games simulated for the proof including $G_{0},G_{1},G_{2},G_{3},G_{4},G_{5}$ with increasing success probabilities of 𝒜 in attacking the protocol. The ultimate goal of 𝒜 is to retrieve the bit b using the *Test* query after each of the games finishes. $Pr[S_{i}]$ is denoted as success probabilities, in which $E_{f}$ (f=0,1,2,3,4,5) are events in respective games. I set a simulator Ş to play the role of the challenge Ç in the games. □

Game $G_{0}$: This is the starting game, which is identical to the real protocol in the RoR model. Ş tosses the coin b and the game is started. We obtain

(2) $${Adv}_{Ç}^{IoLTHC}=\left|2\mathrm{Pr}\left[E_{0}\right]-1\right|$$

Game $G_{1}$: This game executes all queries that are specified in the model. The queries are simulated in Table 4 in accordance with rules of my proposed protocol. In this way, $G_{1}$ creates three lists, namely, $L_{h}$, $L_{r}$, and $L_{t}$. Since $G_{0}$ and $G_{1}$ are indistinguishable, we have

(3) $$\mathrm{Pr}\left[E_{1}\right]=\mathrm{Pr}\left[E_{0}\right]$$

Game $G_{2}$: In this game, the author considers collision probabilities of hash oracle queries and random oracle queries for all transcripts conveyed between $P_{i}$ and $S_{j}$. Based on a property of the birthday paradox, the probability of the hash queries is at most $\frac{{q_{h}}^{2}}{2^{L_{h}+1}}$. During login and authentication procedures of the protocol, $P_{i}$ and $S_{j}$ generate three random numbers $\left\{a_{i},b_{j},c_{j}\right\}$ for constructing two messages $\left\{{DID}_{i},R_{i},{Auth}_{i},T_{p}\right\}$ and $\left\{\left[x_{1},x_{2},\ldots ,x_{n}\right],Y_{j},{Auth}_{j},T_{s}\right\}$. Its total collision probability is $\frac{{\left(q_{s}+q_{e}\right)}^{3}}{2^{L_{r}+1}}$. Due to the indistinguishability between $G_{1}$ and $G_{2}$, the following equation is obtained:

(4) $$|\mathrm{Pr}\left[E_{2}\right]-\mathrm{Pr}\left[E_{1}\right]|\leq \frac{{\left(q_{s}+q_{e}\right)}^{3}}{2^{L_{r}+1}}+\frac{{q_{h}}^{2}}{2^{L_{h}+1}}$$

Game $G_{3}$: $G_{3}$ is similar to $G_{2}$, but *Send*(Ç, m) queries are made for each communicated message. This game consists of two cases consistent with two messages sent by $P_{i}$ and $S_{j}$.

*+ Case 1*: Query *Send*($S_{j},m_{1}$) is simulated in this case. Messages $m_{1}$ is computed from three values ${ID}_{i}^{*}⊕y_{Mi},a_{i}\cdot P,h(x_{Ti}||x_{Mi}||T_{p})\in L_{h}$. To lauch the attack, the hash value PB should also be revealed to 𝒜. It results in a total probability of 4$\frac{q_{h}}{2^{L_{h}}}$ in total. Meanwhile, the random number $a_{i}$ included in $m_{1}$ has a probability at most of $\frac{q_{s}}{2^{L_{r}}}$.

*+ Case 2*: Query *Send*($P_{i},m_{2}$) is executed in this case. To launch the attack, the values $b_{j}⊕h({ID}_{i}^{*}|\left|y_{Ti}^{*}\right||T_{s})$, $h(y_{Ti}^{*}||{ID}_{i}||b_{j}|\left|ck\right)$, $H_{j}=h(b_{j}||T_{s})$, $h(y_{Ti}||T_{s})$, $h^{t_{1}-1}(h(prk||b_{j}))$, and $h^{z-t_{2}}(h(prk||c_{j}))$ containing messages $m_{2}$ should be known to 𝒜. Therefore, its maximum probability is up to 6$\frac{q_{h}}{2^{L_{h}}}$. Random numbers $b_{j},c_{j}$ have a probability of, at most, 2$\frac{q_{s}}{2^{L_{r}}}$.

Since $G_{2}$ and $G_{3}$ are identical when these attacks are absent, we obtain

(5) $$|\mathrm{Pr}\left[E_{3}\right]-\mathrm{Pr}\left[E_{2}\right]|\leq 10\frac{q_{h}}{2^{L_{h}}}+3\frac{q_{s}}{2^{L_{r}}}$$

Game $G_{4}$: Guessing attacks executed by 𝒜 are simulated in this game. The author includes five attack cases, which are described as follows.

*+ Case 1*: 𝒜 runs query *Corrupt*$\left(P_{i},w=1\right)$ to guess ${PW}_{i}$ of $P_{i}$. Next, 𝒜 makes query *Send*($S_{j},m_{1}$) for the attacks. The probability in this case is at most ($C^{\prime }\cdot q_{s}^{s^{\prime }}$).

*+ Case 2*: 𝒜 runs the query *Corrupt*$\left(P_{i},w=3\right)$ to retrieve $B_{i}$ of $P_{i}$. 𝒜 also executes query *Send*($S_{j},m_{1}$) to launch the attack; therefore, the collision probability is up to $max\{q_{s}(\frac{1}{2^{l_{bio}}},\varepsilon_{bio})\}$.

*+ Case 3*: Suppose 𝒜 employs power analysis to successfully retrieve parameters stored in ${SC}_{i}$. Upon *Hash* oracle queries, 𝒜 aims to break the ECDLP to compromise the values ${CID}_{i},PB,a_{i}$ (based on the points $W_{i},R_{i}$, respectively) in order to impersonate $P_{i}$. The probability in this case is at most $2q_{h}{Adv}_{𝒜}^{ECDLP}(t_{𝒜})$.

*+ Case 4*: To trigger MITM attacks or impersonation attacks, 𝒜 runs *Hash* oracle queries that break the ECCDHP assumption to compromise the point $M_{i}=a_{i}\cdot prk\cdot P$ given the points $R_{i}=a_{i}\cdot P$ and puk=prk·P. Its maximum collision probability is up to $q_{h}{Adv}_{𝒜}^{ECCDHP}(t_{𝒜})$.

*+ Case 5*: To trigger similar attacks, 𝒜 runs *Hash* oracle queries to break the ECFP to compromise two points ${TID}_{i}={CID}_{i}\cdot P$ and PB·P given the point $W_{i}=[{CID}_{i}+PB]P$ (retrieved from ${SC}_{i}$ using power analysis). In this case, the collision probability is at most $q_{h}{Adv}_{𝒜}^{ECFP}(t_{𝒜})$.

Because $G_{3}$ and $G_{4}$ are indistinguishable, we have

(6) $$\left|\mathrm{Pr}\left[E_{4}\right]-\mathrm{Pr}\left[E_{3}\right]\right|\leq max\left\{\left(C^{\prime }\cdot q_{s}^{s^{\prime }}\right),q_{s}\left(\frac{1}{2^{l_{bio}}},\varepsilon_{bio}\right)\right\}+2q_{h}{Adv}_{𝒜}^{ECDLP}(t_{𝒜})\;+q_{h}{Adv}_{𝒜}^{ECCDHP}(t_{𝒜})+q_{h}{Adv}_{𝒜}^{ECFP}(t_{𝒜})$$

Game $G_{5}$: The author simulates attack scenarios on the forward secrecy property in this last game. Based on the current transcripts, *Execute*, *Send*, and *Hash* oracle queries are executed to retrieve group session keys generated by the old transcripts. The ECDLP assumption, ECCDHP assumption, and ECFP assumption are included in the simulation. To this end, the *Test* query is made to return the session key to 𝒜. To launch the attacks, 𝒜 has to at least break the ECDLP two times in a row, to break the ECCDHP one time, or to break the ECFP one time; therefore, the following equation is obtained:

(7) $$|\mathrm{Pr}\left[E_{5}\right]-\mathrm{Pr}\left[E_{4}\right]|\leq 2q_{h}(q_{s}+q_{e}){Adv}_{𝒜}^{ECDLP}(t_{𝒜})\;+q_{h}(q_{s}+q_{e}){Adv}_{𝒜}^{ECCDHP}(t_{𝒜})\;+q_{h}(q_{s}+q_{e}){Adv}_{𝒜}^{ECFP}(t_{𝒜})$$

After all games are made, the bit $b^{\prime }$ is guessed upon the probability of the *Test* query below:

(8) $$\mathrm{Pr}\left[E_{5}\right]=\frac{1}{2}$$

Applying property of the triangular inequality and results of Equations (3)–(8), we have

(9) $$|\mathrm{Pr}\left[E_{0}\right]-\frac{1}{2}|=|\mathrm{Pr}\left[E_{1}\right]-\mathrm{Pr}\left[E_{5}\right]|\;\leq \left|\mathrm{Pr}\left[E_{1}\right]-\mathrm{Pr}\left[E_{2}\right]\right|\;+\left|\mathrm{Pr}\left[E_{2}\right]-\mathrm{Pr}\left[E_{3}\right]\right|\;+\left|\mathrm{Pr}\left[E_{3}\right]-\mathrm{Pr}\left[E_{4}\right]\right|\;+\left|\mathrm{Pr}\left[E_{4}\right]-\mathrm{Pr}\left[E_{5}\right]\right|$$

Applying Equations (2)–(9), the following result is achieved:

(10) $$\frac{1}{2}{Adv}_{Ç}^{IoLTHC}=|\mathrm{Pr}\left[E_{0}\right]-\frac{1}{2}|\;\leq \frac{{\left(q_{s}+q_{e}\right)}^{3}}{2^{L_{r}+1}}+\frac{{q_{h}}^{2}}{2^{L_{h}+1}}+10\frac{q_{h}}{2^{L_{h}}}+3\frac{q_{s}}{2^{L_{r}}}+max\left\{\left(C^{\prime }\cdot q_{s}^{s^{\prime }}\right),q_{s}\left(\frac{1}{2^{l_{bio}}},\varepsilon_{bio}\right)\right\}\;+2q_{h}\left(q_{s}+q_{e}+1\right){Adv}_{𝒜}^{ECDLP}\left(t_{𝒜}\right)+q_{h}(q_{s}+q_{e}+1){Adv}_{𝒜}^{ECCDHP}(t_{𝒜})\;+q_{h}(q_{s}+q_{e}+1){Adv}_{𝒜}^{ECFP}(t_{𝒜})$$

Multiplying two sides of Equation (10) with a factor of 2, we can easily obtain the following final result:

(11) $${Adv}_{Ç}^{IoLTHC}\leq \frac{{\left(q_{s}+q_{e}\right)}^{3}+6q_{s}}{2^{L_{r}}}+\frac{{q_{h}}^{2}+20q_{h}}{2^{L_{h}}}+2max\left\{\left(C^{\prime }\cdot q_{s}^{s^{\prime }}\right),q_{s}\left(\frac{1}{2^{l_{bio}}},\varepsilon_{bio}\right)\right\}\;+4q_{h}\left(q_{s}+q_{e}+1\right){Adv}_{𝒜}^{ECDLP}\left(t_{𝒜}\right)+2q_{h}(q_{s}+q_{e}+1){Adv}_{𝒜}^{ECCDHP}(t_{𝒜})\;+2q_{h}(q_{s}+q_{e}+1){Adv}_{𝒜}^{ECFP}(t_{𝒜})$$

As can be seen, Equation (1) and Equation (11) are consistent. Hence, Theorem 1 is claimed and the proposed protocol is proven to be secure, as ${Adv}_{Ç}^{IoLTHC}$ is a completely negligible advantage.

## 7. Performance Evaluation and Comparison

This section provides a detailed performance evaluation and presents a comparative study on multiple aspects of the protocols, including security properties and functionalities, computation overhead, and communication overhead.

### 7.1. Security Properties and Functionalities

The author provides the results of a comparison of security properties and functionalities of different works discussed in Section 2.2, which are tabulated in Table 5. As can be seen, the proposed protocol provides more functionality and achieves more security properties compared to the others. Especially notable is that only the proposed work introduces a 6G-aided group-based dynamic U-healthcare application. In addition, this work is the first to employ a sequencer to directly store user’s registered credentials as well as use it as a separate factor for the authentication in a key agreement protocol.

### 7.2. Computation Overhead

**Figure 7:**
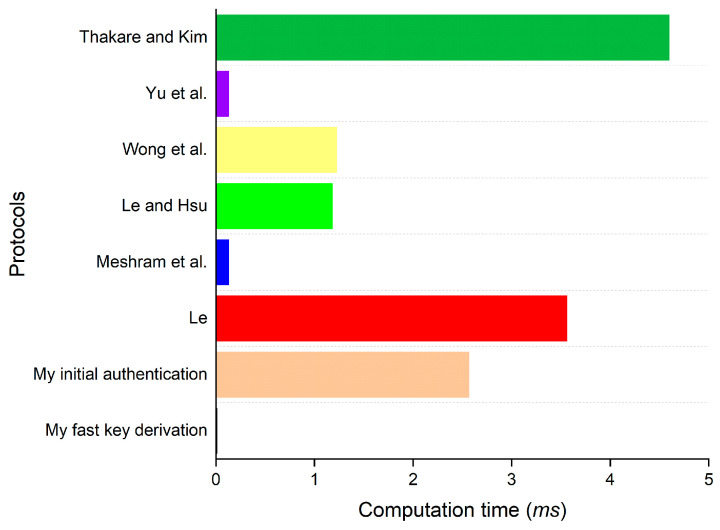
Graphical description of the comparison of computation overhead [28,29,30,31,36,37].

Six of the eleven existing works above, which are the most relevant to the proposed approach, are included for evaluating the computation overhead and communication overhead. To estimate the overhead, the author calculates the running time of all cryptographic operations in the login and authentication phase of each protocol. Since XOR operations are so fast, its running time is assumed to be negligible. For simplicity, the computing times of a traditional one-way hash function and a biohash function are also considered to be similar, as the difference between them is too small [29,32]. The running time of each cryptographic operation used in the evaluation is tabulated in Table 6. The comparative results of the computation overhead evaluation are described in Table 7 and Figure 7. Giving the support of far fewer functional properties (specified in Table 5), the protocols of Yu et al. [29], Wong et al. [30], Le and Hsu [31], and Meshram et al. [36] incur less computing cost compared to that in the initial authentication procedure of the proposed work. However, overhead consumed in the fast key derivation of the proposed work is less than that of all other protocols, which makes it become the most efficient procedure.

**Figure 8:**
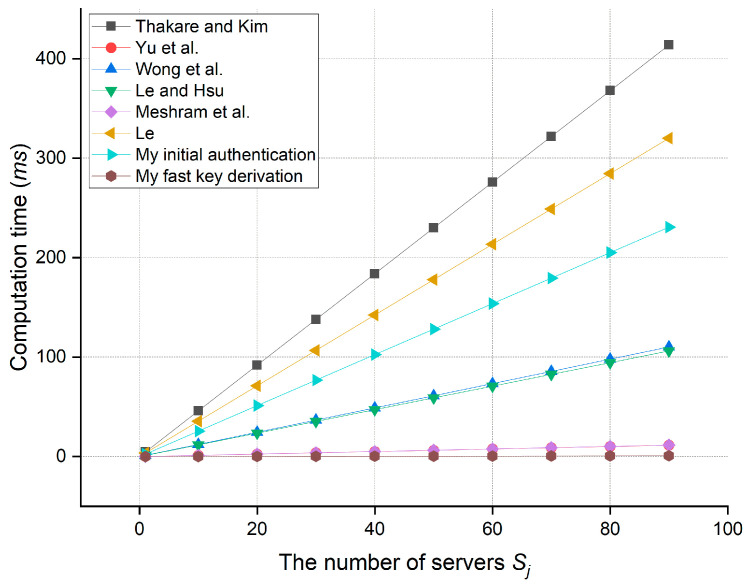
Computational comparison when the number of servers gradually increases [28,29,30,31,36,37].

Apart from that, the author considers a scenario in which multiple $S_{j}$ provide services to a single $P_{i}$. Here, the SD-SSO function is helpful since it allows $P_{i}$ to enjoy multiple services using a single set of credentials for the login. The SD-SSO also save a little bit of computing cost as its operations, including $\sigma =\varepsilon_{i}⊕h({ID}_{i}^{*}|\left|{PW}_{i}^{*}\right||h_{bio}(B_{i}^{*}))$, ${PB}^{*}=h({ID}_{i}^{*}|\left|{PW}_{i}^{*}\right||h_{bio}(B_{i}^{*})||\sigma )$ and $V≟h\left({ID}_{i}^{*}\left|\left|{PB}^{*}\right|\right|\sigma \right)$, only need to be executed once before the communications with multiple $S_{j}$. According to the result depicted in Figure 8, when the number of servers $S_{j}$ increases, both procedures of the proposed protocol (especially the fast key derivation) incur less and less overhead compared with that of the others. Furthermore, due to the group key, $S_{j}$ in the proposed architecture only needs to encrypt health data once before sending it to all $P_{i}$ while $S_{j}$ in the other works (except Le and Hsu [31]) must encrypt the same data multiple times, which results in redundant computation costs. Moreover, the patients in those works are not able to directly communicate with each other without a common key. As a matter of fact, the proposed group communication solution in this work is the best fit for group-based U-healthcare services.

### 7.3. Communication Overhead

**Figure 9:**
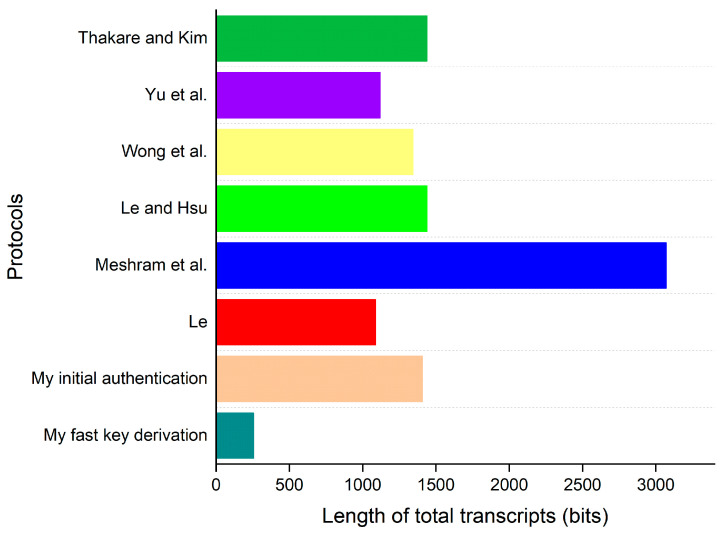
Graphical description of the comparison of communication overhead [28,29,30,31,36,37].

In this evaluation, communication overhead includes the number of communication rounds and total length of all transmitted transcripts. Some parameters used for evaluating the overhead are provided in Table 8. In the initial authentication procedure of the proposed protocol, the transcripts of two communication rounds include $\left\{{DID}_{i},R_{i},{Auth}_{i},T_{p}\right\}$ and $\left\{\left[x_{1},x_{2},\ldots ,x_{n}\right],Y_{j},{Auth}_{j},T_{s}\right\}$. For a fair comparison, $\left\{\left[x_{1},x_{2},\ldots ,x_{n}\right],Y_{j},{Auth}_{j},T_{s}\right\}$ should contain parameters of a single patient, which only results in a single value x in the transcript. $\left\{{DID}_{i},R_{i},{Auth}_{i},T_{p}\right\}$ and $\left\{x,Y_{j},{Auth}_{j},T_{s}\right\}$ consume a length of (160 bits + 320 bits + 160 bits + 32 bits) and (384 bits + 160 bits + 160 bits + 32 bits), respectively; the total length is (672 bits + 736 bits) = 1408 bits. Similarly, overhead values of all protocols are calculated and provided in Table 9. Figure 9 further provides a graphical description of the comparison. We can observe that the proposed protocol incurs less overhead than the works of Thakare and Kim [28], Le and Hsu [31], and Meshram et al. [36]. Due to providing the support of more functionality, the author’s work consumes more costs compared to that of Yu et al. [29], Wong et al. [30], and Le [37]. Furthermore, when the proposed work executes the fast key-derivation process, its communication only incurs 256 bits (the length of $C_{d}$) and only one communication round. As a result, it is the most efficient out of all the protocols.

## 8. Conclusions

In this article, the author has proposed a group-based patient-authenticated key distribution protocol for 6G-aided dynamic U-healthcare services enabled by real-time mobile DNA sequencing. Seamless communications are provided by 6G technology regardless of patients’ geographical locations. Sharing mobile DNA data for rapid analysis is a good solution for facilitating drug and vaccine development, which is one of the important concerns in the public health sector. Group service helps in improving medical treatments efficiently and promoting the use of smart health with more people participating. Patients in a healthcare group are allowed to securely connect with the service provider or with each other using a common group key generated from the protocol for the specific purposes of dynamic services. The group key generation process is protected by a three-factor authentication mechanism along with an efficient SD-SSO solution. Since all registered credentials are stored on a separate sequencer, the proposed work can enable service availability on multiple mobile devices. It is also able to facilitate a truly patient-centered service upon storing traceable information in the server database. Security analysis of the proposed protocol is presented using well-known verification tools, namely, the RoR model and BAN logic. A semantic discussion is also provided to further indicate its resistance to multiple security attacks. A detailed performance analysis of computation and communication overhead shows that the proposed approach consumes a rational cost compared to predecessor works.

In future works, performance of the initial authentication procedure can be further improved by including more lightweight cryptographic operations in the protocol. Another patient authentication scheme with a new architecture model where multiple external doctors serving as data users join the healthcare processes will be considered. The author will also consider a new design of attribute-based access control for securing cloud-based U-healthcare services in IoLT networks.

## Figures and Tables

**Table 1:** Notations used in the proposed approach.

Notation Used in the Protocol	Explanation
$S_{j}$	The $j^{th}$ server
$P_{i}$	The $i^{th}$ patient
${prk}_{j},{puk}_{j}$	Private key, public key of $S_{j}$
$P_{\left(x,y\right)}$	Basic point on the curve Ep(a,b) with two coordinates x and y
${ID}_{i}$	Identity of $P_{i}$
${PW}_{i}$	Password of $P_{i}$
$B_{i}$	Biometrics of $P_{i}$
${MD}_{i}$	Mobile device of $P_{i}$
${SD}_{i}$	Sequencing device (sequencer) of $P_{i}$
T	Timestamp
||	Concatenating operation
⊕	Exclusive-or (XOR) operation
$h\left(\cdot \right),h_{bio}(\cdot )$	One-way hash function, biohash function
${SE}_{k}\left(\cdot \right),{SD}_{k}\left(\cdot \right)$	Symmetric encryption, symmetric decryption using a key *k*
${\left[\cdot \right]}_{{SD}_{i}}$	Storing parameters in ${SD}_{i}$
𝒜	Adversary

**Table 2:** Notations used in the analysis with BAN logic.

Notations Used in the BAN	Explanation
*X* |≡ *M*	*X* believes a statement *M*
*X* ⊲ *M*	*X* sees the statement *M*
*X* |~ *M*	*X* once said the statement *M*
*X* ⟹ *M*	*X* has jurisdiction over the statement *M*
(*M*, *N*)	*M* or *N* is one part of the formula (*M*, *N*)
${\left\langle M\right\rangle }_{N}$	The statement *M* is combined with the formula *N*
#(*M*)	The formula *M* is fresh, meaning it has not been sent in any previous messages
$X\overset{K}{\Leftrightarrow }Y$	Formula *K* is a secret known only by *X* and *Y*; only *X* and *Y* can use *M* to authenticate each other
$X\overset{G}{↔}Y$	Value *G* is known only to *X* and *Y*; it is used for their communication

**Table 3:** Notations used in the security proof with RoR Model.

Notations	Explanation
$l_{h}$	Size of a hash value
$l_{r}$	Size of a random number
$l_{bio}$	Size of a biometric value
$q_{h}$	Total hash oracle queries
$q_{s}$	Total *Send* queries
$q_{e}$	Total *Execute* queries
$L_{h}$	List of hash oracle outputs
$L_{r}$	List of random oracle results
$L_{t}$	List of transcripts conveyed between $P_{i}$ and $S_{j}$
$\varepsilon_{bio}$	Biometric false-positive probability
$C^{\prime },s^{\prime }$	*Zipf* parameters

**Table 4:** All queries executed in the RoR model.

*Hash* query is executed as follows, where $m_{i}$ are messages. If the record ($m_{i}$, $h(m_{i})$) is found in the list $L_{h}$, return $h(m_{i})$; if not, choose $h^{\prime }\left(m_{i}\right)\in Z_{p}^{*}$ and write ($m_{i}$, $h^{\prime }\left(m_{i}\right)$) to $L_{h}$; the list $L_{r}$ is created by a similar procedure.
*Reveal*(Ç) query is executed by a simple procedure as follows. Once Ç is in an Accept state, a session key formed by Ç is returned.
*Test*(Ç) query is executed as follows. Ç tosses the coin b. If b=1, the query returns an available key ${gk}_{t}$; otherwise, it returns a random number.
*Corrupt*($P_{i},w$) query is executed as follows. If w=1, the query outputs password ${PW}_{i}$. If w=2, the query outputs parameters stored in ${SD}_{i}$. If w=3, the query outputs biometrics $B_{i}$.
*Execute*($P_{i}$, $S_{j}$) query is executed in succession with execution of *Send*(Ç, $m_{i}$) query. It is presented as follows. $P_{i}$ sends $m_{1}$ to $S_{j}$ and $S_{j}$ sends $m_{2}$ to $P_{i}$. we have $<{ID}_{i}^{*}⊕y_{Mi},a_{i}\cdot P,h\left(x_{Ti}\left|\left|x_{Mi}\right|\right|T_{p}\right),T_{p}>$ ← *Send*($P_{i}$, *start*), $<\left[x_{1},x_{2},\ldots ,x_{n}\right],b_{j}⊕h({ID}_{i}^{*}|\left|y_{Ti}^{*}\right||T_{s}),h(y_{Ti}^{*}||{ID}_{i}||b_{j}|\left|ck\right),T_{s}>$ ← *Send*($S_{j}$, $<{ID}_{i}^{*}⊕y_{Mi},a_{i}\cdot P,h\left(x_{Ti}\left|\left|x_{Mi}\right|\right|T_{p}\right),T_{p}>$) At last, $m_{1}=<{ID}_{i}^{*}⊕y_{Mi},a_{i}\cdot P,h\left(x_{Ti}\left|\left|x_{Mi}\right|\right|T_{p}\right),T_{p}>$ and $m_{2}=<\left[x_{1},x_{2},\ldots ,x_{n}\right],b_{j}⊕h({ID}_{i}^{*}|\left|y_{Ti}^{*}\right||T_{s}),h(y_{Ti}^{*}||{ID}_{i}||b_{j}|\left|ck\right),T_{s}>$ are returned.
Based on the logical procedure of the protocol, the *Send* query is simulated as follows. 𝒜 runs *Send*($P_{i}$, *start*) query and Ç replies to 𝒜 as follows. Ç computes $M_{i}=a_{i}\cdot puk=(x_{Mi},y_{Mi})$, $R_{i}=a_{i}\cdot P$, $c_{i,m22}=y_{i,m2}*r_{i,m1}mod\;p$, ${TID}_{i}=W_{i}-{PB}^{*}\cdot P=\left(x_{Ti},y_{Ti}\right)$, and ${Auth}_{i}=h(x_{Ti}||x_{Mi}||T_{p}$) and outputs $m_{1}=<{ID}_{i}^{*}⊕y_{Mi},R_{i},h\left(x_{Ti}\left|\left|x_{Mi}\right|\right|T_{p}\right),T_{p}>$. 𝒜 runs *Send*($S_{j}$, $<{ID}_{i}^{*}⊕y_{Mi},R_{i},h\left(x_{Ti}\left|\left|x_{Mi}\right|\right|T_{p}\right),T_{p}>$) query and Ç replies to 𝒜 as follows. Ç checks $T_{p}$; computes $M_{i}^{*}=prk\cdot R_{i}=(x_{Mi}^{*},y_{Mi}^{*})$ and ${ID}_{i}^{*}={DID}_{i}⊕y_{Mi}^{*}$; checks ${CID}_{i}$; computes ${TID}_{i}^{*}$ and ${Auth}_{i}$; computes ${TB}_{1}=h^{t_{1}-1}(h(prk||b_{j}))$, ${TB}_{2}=h^{z-t_{2}}(h(prk||c_{j}))$, $Y_{j}=b_{j}⊕h({ID}_{i}^{*}|\left|y_{Ti}^{*}\right||T_{s})$, $ck=({TB}_{1}||{TB}_{2}||t_{1}||t_{2})$, ${Auth}_{j}=h(y_{Ti}^{*}||{ID}_{i}||b_{j}|\left|ck\right)$, and $H_{j}=h(b_{j}|\left|T_{s}\right)$; and generates $\left[x_{1},x_{2},\ldots ,x_{n}\right]$ from $H_{j},y_{Ti},ck$. Ç terminates the session if one of the above checks does not hold. Otherwise, Ç outputs $m_{2}=<\left[x_{1},x_{2},\ldots ,x_{n}\right],b_{j}⊕h({ID}_{i}^{*}|\left|y_{Ti}^{*}\right||T_{s}),h(y_{Ti}^{*}||{ID}_{i}||b_{j}|\left|ck\right),T_{s}>$. The session key $S_{j}$ obtains is ${gk}_{t}=h(h^{t-1}(h(prk|\left|b_{j}\right))||h^{z-t}\left(h(prk||c_{j})\right))$. 𝒜 runs *Send*($P_{i}$, $<\left[x_{1},x_{2},\ldots ,x_{n}\right],b_{j}⊕h({ID}_{i}^{*}|\left|y_{Ti}^{*}\right||T_{s}),h(y_{Ti}^{*}||{ID}_{i}||b_{j}|\left|ck\right),T_{s}>$) query and Ç replies to 𝒜 as follows. Ç checks $T_{s}$; computes $b_{j},H_{j},{ck}^{*}$ based on some related parameters; and checks ${Auth}_{j}$. If one of the checks does not hold, Ç terminates the session; otherwise, a session key ${gk}_{t}=h(h^{t-t_{1}}({TB}_{1})||h^{t_{2}-t}\left({TB}_{2}\right))$ is established, and the session is completed.

**Table 5:** Security properties and functionalities of different protocols.

Attributes	[21]	[23]	[25]	[26]	[27]	[28]	[29]	[30]	[31]	[36]	[37]	Mine
Resists replay attacks	O	O	O	O	O	O	O	O	O	O	O	O
Resists MITM attacks	O	O	O	O	O	O	O	O	O	O	O	O
Resists online password guessing attacks	–	O	O	O	O	O	O	O	O	O	O	O
Resists offline password guessing attacks	–	O	O	O	O	O	O	O	O	O	O	O
Resists impersonation attacks	O	O	O	O	O	O	O	O	O	O	O	O
Resists lost sequencer or smart card attacks	O	O	–	O	–	–	O	X	X	O	O	O
Resists desynchronization attacks	O	O	O	O	O	O	O	X	O	X	O	O
Resists privileged insider attacks	O	O	O	O	O	O	O	O	O	O	O	O
Resists DoS attacks	O	O	O	O	O	O	O	O	O	O	O	O
Provides mutual authentication	O	O	O	O	O	O	O	O	O	O	O	O
Provides user anonymity	O	O	O	O	O	O	O	X	X	O	O	O
Provides user untraceability	O	O	O	O	O	X	O	O	O	X	O	O
Provides message unlinkability	O	O	O	O	O	X	O	O	O	X	O	O
Provides perfect forward secrecy	O	O	O	O	O	O	O	O	O	O	O	O
Provides perfect backward secrecy	O	O	O	O	O	O	O	O	O	O	O	O
Provides password update	–	O	O	X	O	O	O	X	X	O	O	O
Provides biometrics update	–	–	–	–	–	–	O	X	X	–	O	O
Provides three-factor authentication	X	X	X	X	X	X	O	O	O	X	O	O
Provides SD-SSO	X	X	X	X	X	X	X	X	X	X	X	O
Provides mathematics-based security proof	X	X	O	O	O	X	O	X	O	X	O	O
Provides group-based dynamic services	X	X	X	X	X	X	X	X	X	X	X	O
Provides LOC-based U-healthcare application	X	X	X	X	X	X	X	X	X	X	O	O
Supports patient-centric service	O	X	O	X	O	O	X	O	O	O	X	O

“O”: the protocol achieves a specific attribute; “X”: the protocol does not achieve a specific attribute; “–”: A specific attribute is not available in the protocol.

**Table 6:** Time estimation of each cryptographic operation [44,45].

Notation	Operation	Computation Time (ms)
$T_{H}$	Hash function	≈0.00069
$T_{PA}$	EC point addition	≈0.0069
$T_{PM}$	EC point multiplication	≈0.508
$T_{SED}$	Symmetric encryption or decryption	≈0.00054
$T_{M}$	Modular squaring	≈0.00069
$T_{QR}$	Square root module 𝑁	≈1.169
$T_{CM}$	Chebyshev chaotic polynomial mapping	≈0.02881

**Table 7:** Comparison of computation overhead.

Protocols		$P_{i}$		$S_{j}$		Total Computation Time (ms)
		Computation Complexities	Computation Time (ms)	Computation Complexities	Computation Time (ms)	
Thakare and Kim [28]		$2T_{H}$$+T_{PA}$ + $4T_{PM}$	≈2.04028	$6T_{H}$ + $2T_{PA}$ + $5T_{PM}$	≈2.55794	≈4.59822
Yu et al. [29]		$10T_{H}$ + $2T_{CM}$	≈0.06452	$8T_{H}$ + $2T_{CM}$	≈0.06314	≈0.12766
Wong et al. [30]		$7T_{H}$ $+T_{SED}$ $+T_{M}$	≈0.04953	$7T_{H}$ $+2T_{SED}$ $+T_{QR}$	≈1.17491	≈1.22444
Le and Hsu [31]		$9T_{H}$ +$T_{SED}$ + $T_{M}$	≈0.00744	$5T_{H}$$+2T_{SED}$ + $T_{QR}$	≈1.17353	≈1.18097
Meshram et al. [36]		$11T_{H}$ + $2T_{CM}$	≈0.06521	$9T_{H}$ +$\;2T_{CM}$	≈0.06383	≈0.12904
Le [37]		$4T_{H}$$+T_{SED}$ + $4T_{PM}$	≈2.03530	$2T_{H}$$+T_{SED}$ + $3T_{PM}$	≈1.52592	≈3.56122
Mine	Initial authentication	$13T_{H}$$+T_{PA}$ + $3T_{PM}$	≈1.53987	$16T_{H}$ + $2T_{PM}$	≈1.02704	≈2.56691
	Fast key derivation	$3T_{H}$ +$T_{SED}$	≈0.00261	$9T_{H}$ +$T_{SED}$	≈0.00675	≈0.00936

**Table 8:** Single length of multiple parameters [44,45].

Parameters	Length (Bits)
Asymmetric encryption or decryption (e.g., Rabin system)	1024
Chebyshev polynomial	1024
Symmetric encryption or decryption	256
Identity	128
Password	128
Biometrics	128
Random number	160
Hash value	160
EC point multiplication	320
Timestamp	32

**Table 9:** Comparison of communication overhead.

Protocols		No. of Communication Rounds	Length of Total Transcripts (Bits)
Thakare and Kim [28]		3	1440
Yu et al. [29]		3	1120
Wong et al. [30]		2	1344
Le and Hsu [31]		2	1440
Meshram et al. [36]		2	3072
Le [37]		2	1088
Mine	Initial authentication	2	1408
	Fast key derivation	1	256

## Data Availability

Not available.
